# Desmoplastic Reaction, Immune Cell Response, and Prognosis in Colorectal Cancer

**DOI:** 10.3389/fimmu.2022.840198

**Published:** 2022-03-22

**Authors:** Naohiko Akimoto, Juha P. Väyrynen, Melissa Zhao, Tomotaka Ugai, Kenji Fujiyoshi, Jennifer Borowsky, Rong Zhong, Koichiro Haruki, Kota Arima, Mai Chan Lau, Junko Kishikawa, Tyler S. Twombly, Yasutoshi Takashima, Mingyang Song, Xuehong Zhang, Kana Wu, Andrew T. Chan, Jeffrey A. Meyerhardt, Marios Giannakis, Jonathan A. Nowak, Shuji Ogino

**Affiliations:** ^1^ Program in Molecular Pathological Epidemiology, Department of Pathology, Brigham and Women’s Hospital, Harvard Medical School, Boston, MA, United States; ^2^ Department of Gastroenterology, Nippon Medical School, Graduate School of Medicine, Tokyo, Japan; ^3^ Department of Medical Oncology, Dana-Farber Cancer Institute, Harvard Medical School, Boston, MA, United States; ^4^ Cancer and Translational Medicine Research Unit, Medical Research Center Oulu, Oulu University Hospital, and University of Oulu, Oulu, Finland; ^5^ Department of Epidemiology, Harvard T.H. Chan School of Public Health, Boston, MA, United States; ^6^ Department of Nutrition, Harvard T.H. Chan School of Public Health, Boston, MA, United States; ^7^ Clinical and Translational Epidemiology Unit, Massachusetts General Hospital, Harvard Medical School, Boston, MA, United States; ^8^ Division of Gastroenterology, Massachusetts General Hospital, Boston, MA, United States; ^9^ Channing Division of Network Medicine, Department of Medicine, Brigham and Women’s Hospital, Harvard Medical School, Boston, MA, United States; ^10^ Department of Immunology and Infectious Diseases, Harvard T.H. Chan School of Public Health, Boston, MA, United States; ^11^ Broad Institute of MIT and Harvard, Cambridge, MA, United States; ^12^ Department of Medicine, Brigham and Women’s Hospital, Harvard Medical School, Boston, MA, United States; ^13^ Cancer Immunology and Cancer Epidemiology Programs, Dana-Farber Harvard Cancer Center, Boston, MA, United States

**Keywords:** cancer-associated fibroblast (CAF), clinical outcomes, host–tumor interaction, lymphocytic reaction, microsatellite instability, molecular pathological epidemiology (MPE), immune response, tumor immune microenvironment

## Abstract

**Background:**

The relationships between tumor stromal features (such as desmoplastic reaction, myxoid stroma, and keloid-like collagen bundles) and immune cells in the colorectal carcinoma microenvironment have not yet been fully characterized.

**Methods:**

In 908 tumors with available tissue among 4,465 incident colorectal adenocarcinoma cases in two prospective cohort studies, we examined desmoplastic reaction, myxoid stroma, and keloid-like collagen bundles. We conducted multiplex immunofluorescence for T cells [CD3, CD4, CD8, CD45RO (PTPRC), and FOXP3] and for macrophages [CD68, CD86, IRF5, MAF, and MRC1 (CD206)]. We used the inverse probability weighting method and the 4,465 incident cancer cases to adjust for selection bias.

**Results:**

Immature desmoplastic reaction was associated with lower densities of intraepithelial CD3^+^CD8^+^CD45RO^+^ cells [multivariable odds ratio (OR) for the highest (vs. lowest) density category, 0.43; 95% confidence interval (CI), 0.29–0.62; P_trend_ <0.0001] and stromal M1-like macrophages [the corresponding OR, 0.44; 95% CI, 0.28–0.70; P_trend_ = 0.0011]. Similar relations were observed for myxoid stroma [intraepithelial CD3^+^CD8^+^CD45RO^+^ cells (P_trend_ <0.0001) and stromal M1-like macrophages (P_trend_ = 0.0007)] and for keloid-like collagen bundles (P_trend_ <0.0001 for intraepithelial CD3^+^CD8^+^CD45RO^+^ cells). In colorectal cancer-specific survival analyses, multivariable-adjusted hazard ratios (with 95% confidence intervals) were 0.32 (0.23–0.44; P_trend_ <0.0001) for mature (vs. immature) desmoplastic reaction, 0.25 (0.16–0.39; P_trend_ <0.0001) for absent (vs. marked) myxoid stroma, and 0.12 (0.05–0.28; P_trend_ <0.0001) for absent (vs. marked) keloid-like collagen bundles.

**Conclusions:**

Immature desmoplastic reaction and myxoid stroma were associated with lower densities of tumor intraepithelial memory cytotoxic T cells and stromal M1-like macrophages, likely reflecting interactions between tumor, immune, and stromal cells in the colorectal tumor microenvironment.

## Introduction

Tumor–host interactions have been recognized as important determinants of cancer progression (1, 2). An antitumor immune response requires coordinated efforts of various cells including T cells and macrophages (1, 2). Evidence indicates that cancer development and progression are influenced by interactions between tumor, immune, and other stromal cells. However, the relationship between immune and other stromal cells in the tumor microenvironment remains to be further studied (3–5).

Desmoplastic reaction to tumor denotes the growth of fibrous connective tissue around tumor cells and has been recognized as a potential prognostic marker for colorectal cancer. It is usually classified into (1) an immature type that is characterized by myxoid stroma composed of basophilic, amorphous extracellular matrix; (2) an intermediate type, defined by hyalinized thick bundles of hypocellular keloid-like collagen; and (3) a mature type that demonstrates neither myxoid stroma nor keloid-like collagen (6–13).

Although a study has shown that the number of CD3^+^ lymphocytes was lower in tumors with immature stroma (11), the relationship of desmoplastic reaction and its morphological components with more detailed immune cell types has not been adequately elucidated. Additionally, the three-tiered classification for desmoplastic reaction is based on a joint evaluation of myxoid stroma and keloid-like collagen, and the relative prognostic significance of each component remains unclear. While ample evidence supports the clinical efficacy of desmoplastic reaction in cases with pT3 and pT4 invasion, several studies, including patients who undergone surgical or endoscopic mucosal resection, have suggested an association of desmoplastic reaction in biopsy specimen with massive invasion into the submucosal layer in pT1 cases (14–17). Therefore, we have included all desmoplastic reaction cases regardless of pT stages.

In this study, we utilized a molecular pathological epidemiology database of 908 colorectal cancer cases among 4,465 cases that had occurred in two U.S.-wide prospective cohort studies. We measured T-cell and macrophage densities using two customized 7-plex immunofluorescence assays. We tested the hypothesis that densities of certain T-cell and macrophage subsets might be inversely associated with immature desmoplastic reaction. In addition, we assessed the prognostic role of desmoplastic reaction, myxoid stroma, and keloid-like collagen bundles as well as their statistical interactions with specific T-cell and macrophage subsets in survival analyses.

## Material and Methods

### Study Population

**Figure 1:**
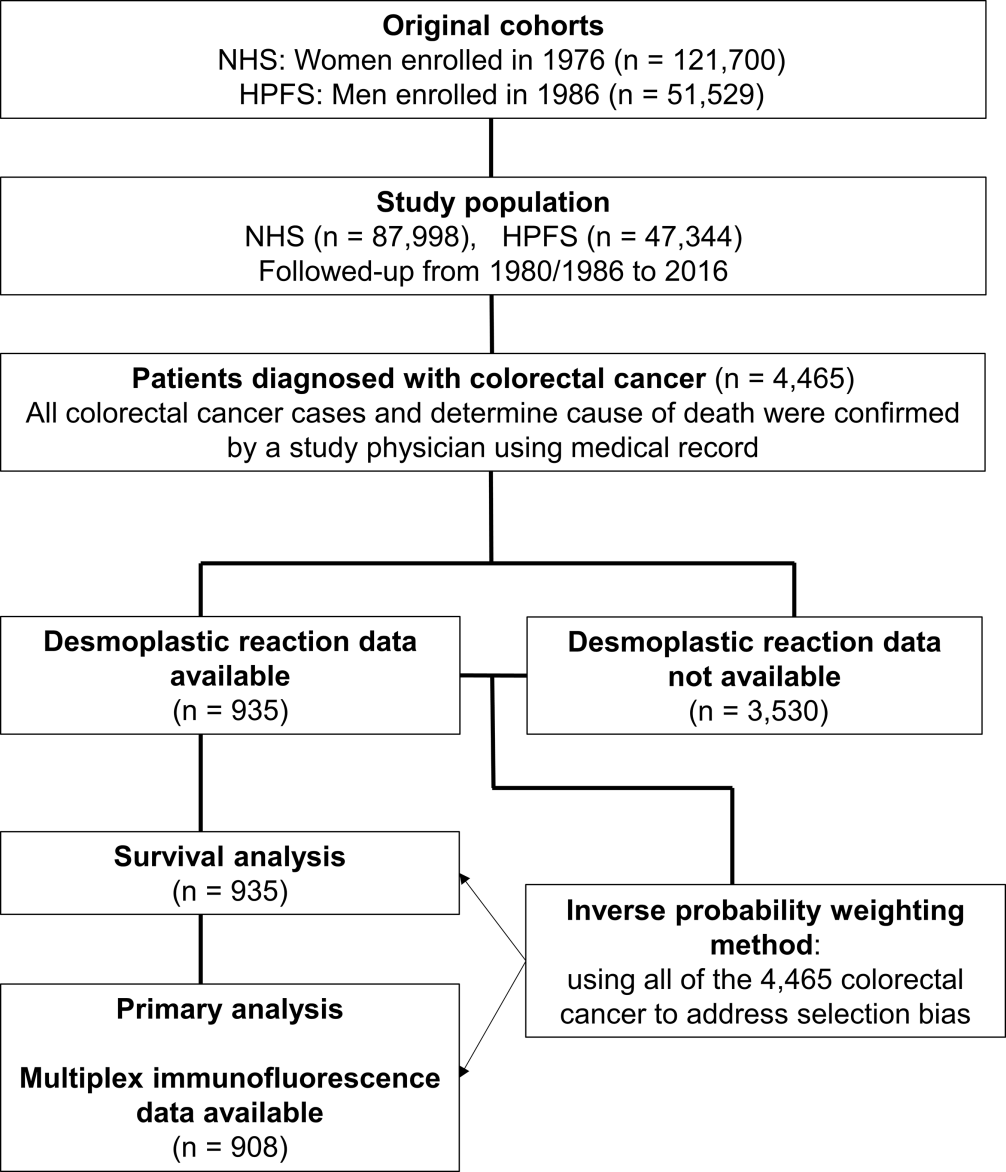
Flow diagram of study population for the analyses with inverse probability weighting. HPFS, Health Professionals Follow-up Study; NHS, Nurses’ Health Study.

The study population base (**Figure 1**) consisted of a total of 173,229 participants of the Health Professionals Follow-up Study (HPFS) (18) and the Nurses’ Health Study (NHS) (19). The participants had been followed over decades *via* biennial questionnaires up to 2014, and a fraction of them (N = 4,465) developed colorectal carcinoma during the follow-up period. Deaths of colorectal cancer patients were ascertained through questionnaire return by next-of-kin and the use of the National Death Index, which also helped us find lethal unreported colorectal cancer cases. Medical record review conducted by a study physician could confirm all colorectal cancer cases and determine cause of death in case of lethal cancer. Formalin-fixed paraffin-embedded (FFPE) tumor tissue blocks were accessed and retrieved from the hospitals where participants had been treated with surgical resection. We utilized all of the 4,465 cases to adjust for selection bias due to tissue data availability (see *Statistical Analyses*). Among the 4,465 patients, histopathological features of desmoplastic reaction were successfully assessed in 935 cases. Among those, we analyzed T-cell and macrophage densities and desmoplastic reaction in colorectal cancer tissue in 908 patients. On the basis of the colorectal continuum model, both colon and rectal carcinomas were included (20, 21). The study population analyzed in this study overlapped with several of our previous studies (22–25), but differed by the number of patients, the tested hypotheses, and new data generated in this study. The study was approved by the institutional review boards of the Brigham and Women’s Hospital and Harvard T.H. Chan School of Public Health (Boston, MA). All study participants provided informed consent. Participants with or without tumor tissue data exhibited no major clinical or demographic differences according to preceding preliminary studies (26–28).

### Immunohistochemistry and Tumor Morphology

**Figure 2:**
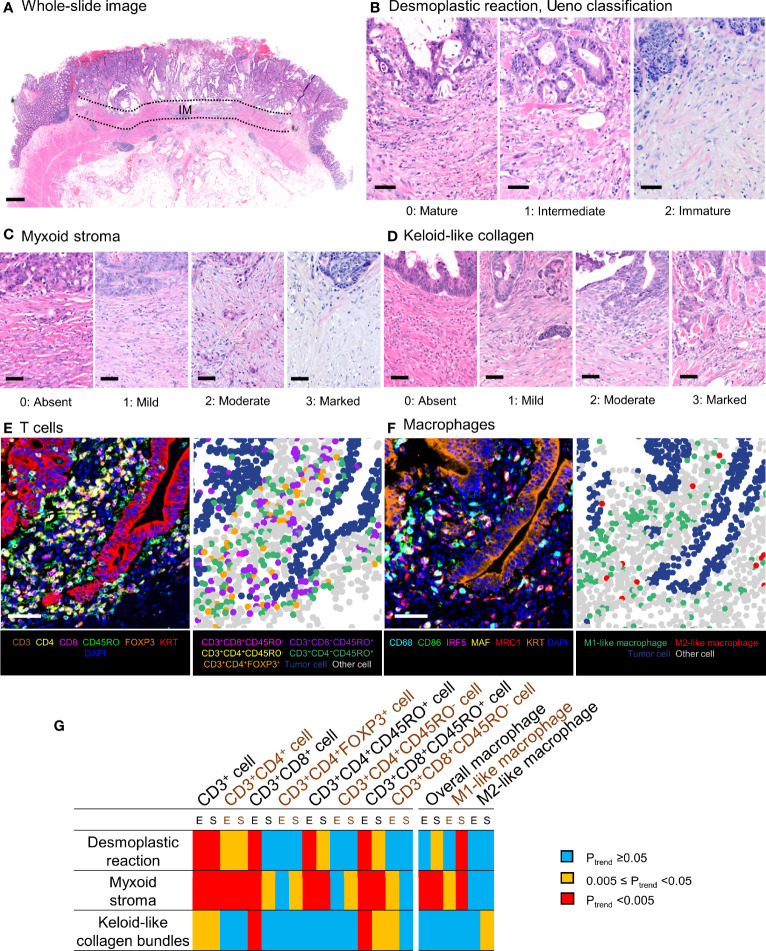
Evaluation of tumor stromal features and T-cell and macrophage infiltrates. Panels **(A–D)** demonstrate representative examples of the tumor stromal features using hematoxylin and eosin–stained sections. **(A)** The stroma was assessed according to the most immature stromal area at the invasive margin (IM) of the tumor. **(B)** Three-tiered Ueno classification of the desmoplastic reaction. **(C)** Four-tiered classification of myxoid stroma. **(D)** Four-tiered classification of keloid-like collagen. Scale bars indicate 1 mm **(A)** or 50 µm **(B–D)**. **(E, F)** Examples of multiplex immunofluorescence images [**(E)** T cells, **(F)** macrophages]. The images, based on simultaneous measurement of the signal intensities of seven fluorophores, were used to identify individual tumor cells, immune cells, and other cells and further classify them using pathologist-supervised machine learning algorithms. Scale bars indicate 50 µm **(E, F)**. **(G)** A matrix of P_trend_ values in multivariable logistic regression analyses to assess the associations of T-cell and macrophage densities in tumor intraepithelial or stromal regions with desmoplastic reaction and its components with inverse probability weighting. E, tumor intraepithelial region; IM, invasive margin; S, tumor stromal region.

Desmoplastic reaction was assessed using hematoxylin and eosin–stained tissue sections according to the three-tiered scale [mature (0), intermediate (1), and immature (2)] as described by Ueno et al. (5–11, 13, 29–31). In short, desmoplastic reaction was regarded as immature if myxoid changes were present in fibrotic stroma regardless of keloid-like collagen. Otherwise, it was classified into intermediate if stroma contained keloid-like collagen but no myxoid changes, or mature if stroma contained neither keloid-like collagen nor myxoid changes. In addition, myxoid stroma and keloid-like collagen bundles were separately assessed using four-tiered scales [absent (0), mild (1), moderate (2), and marked (3)] (**Figure 2**). The myxoid stroma feature was classified as absent if stroma was composed of dense fibrotic tissue; mild if there was a mildly edematous and loose stromal appearance; moderate if there was loose, edematous, and pale-to-lightly basophilic stroma; or marked if there was prominently loose and pale-to-lightly basophilic stroma. The keloid-like collagen feature was classified into absent if there were no thick collagen bundles; mild if there were slightly thickened but not keloid-like collagen bundles; moderate if there were thick hyalinized bundles comprised of hypocellular eosinophilic hyalinized collagen; or marked if there were prominent and abundant keloid-like collagen bundles. These definitions were also transferred to a visual analog scale (**Figure 2**). These evaluations were conducted using a tumor slide containing the deepest level of invasion according to a previous study (32), and the final assessment was based on a single 10× objective field at the invasive front with the most immature stroma. If two or more patterns were present in this field, the scoring was based on dominant characteristics, although immune cell populations were assessed in all fields regardless of the pattern of maturity. A single pathologist (JV) assessed all cases blinded to other data. A second pathologist (MZ) independently reviewed 135 cases, and the weighted kappa values between the two pathologists were 0.52 for desmoplastic reaction (P <0.0001), 0.57 for myxoid stroma (P <0.0001), and 0.40 for keloid-like collagen bundles (P <0.0001). A single pathologist (SO), blinded to other data, categorized tumor differentiation into well to moderate vs. poor (>50% vs. ≤50% glandular area, respectively) and four components of lymphocytic reaction to tumors (tumor-infiltrating lymphocytes, intratumoral periglandular reaction, peritumoral lymphocytic reaction, and Crohn’s-like lymphoid reaction) as negative/low (0) vs. intermediate (1+) vs. high (2+) (33).

### Analyses of T Cells and Macrophages in Tumor

Tissue microarrays were created using two to four tumor cores, selected to best represent overall tumor morphology (34, 35). The invasive margin or the areas utilized in the evaluation of desmoplastic reaction were not specifically sampled into the tissue microarrays. Two customized multiplex immunofluorescence assays were employed to determine patterns of expression of different T-cell markers [CD3, CD4, CD8, CD45RO (*PTPRC*), and FOXP3] as well as the epithelial cell marker KRT at the same time (panel 1). A second marker panel consisting of macrophage markers [CD68, CD86, IRF5, MAF, and MRC1 (CD206)], again together with KRT (panel 2), was also created, as we have previously published (22, 23, 36) using standardized protein nomenclature recommended by the expert panel (37). Tissue microarray core digital images were acquired with an automated multispectral imaging system (Vectra 3.0, Akoya Biosciences, Hopkinton, MA) at a magnification of x200. Supervised machine learning algorithms (inForm 2.4.1, Akoya Biosciences) were employed to analyze images using tissue segmentation (tumor epithelium, stroma, and other), cell segmentation (nuclear, cytoplasmic, and surface membrane compartments), and cell phenotyping algorithms built in the software (**Figure 2**). We investigated the coexpression patterns of CD3, CD4, CD8, CD45RO, and FOXP3 in each T cell and classified T-cell phenotypes as follows: CD3^+^CD8^+^ cytotoxic T cells, CD3^+^CD4^+^ helper T cells, CD3^+^CD4^+^FOXP3^+^ regulatory T cells, CD3^+^CD4^+^CD45RO^+^ memory T cells, and CD3^+^CD8^+^CD45RO^+^ memory T cells. The inForm software used multinomial logistic regression in phenotype classification. For each cell [detected based on settings determined by study pathologists (JB for T-cell data; JV for macrophage data) such as size thresholds and DAPI intensity thresholds], inForm calculated hundreds of features, including morphological features (such as area and compactness) and texture features (such as Haralick features and spatial frequency measurements). Examples of different cell phenotypes (around 50 per phenotype) were manually annotated by the study pathologists. The software then used lasso regularization with cross-validation to select features and create models for the task. After the models showed satisfactory performance (based on visual examination by study pathologists in around 100 training images, including tumors with various morphologies), they were applied to all study images. The images were reviewed by study pathologists to confirm adequate performance of the classifiers and exclude unrepresentative images. Data were acquired at the single-cell level, and the presence of subsets of T cells and macrophages was quantified in the epithelial and stromal regions of the tumor, as we described earlier (22, 23, 36). Macrophages were characterized using an M1:M2 index of polarization defined by the levels of expression of two M1-polarization markers (CD86, IRF5) and two M2-polarization markers (MAF, MRC1) according to the formula (CD86×IRF5)/(MAF×MRC1) (23). Thus, a higher M1:M2 value represents a greater degree of M1-polarization. For all macrophages identified in the tumor images, the 30% with the highest polarization indices were considered M1-like, whereas those with the lowest 30% were allocated to M2-like for the purpose of this analysis, as we have previously published (23). For each cell subset, cases were classified into quartile categories (C1–C4) if there were ≤25% of cases with zero density. If there were >25% of cases with zero density for a specific cell type, these zero-density cases were grouped together (C1 category), and the remaining (nonzero) cases were divided into tertials (C2–C4). For a binary categorization, T-cell and macrophage densities were categorized as low vs. high based on the median value if the median was above zero; otherwise, as low (zero) vs. high (nonzero).

### Analyses of Tumor Molecular Characteristics

Genomic DNA was extracted from FFPE colorectal carcinoma tissue blocks. Ten microsatellite markers (D2S123, D5S346, D17S250, BAT25, BAT26, BAT40, D18S55, D18S56, D18S67, and D18S487) were assessed by PCR in order to determine the microsatellite instability (MSI) status, whereby the presence of ≥30% of these markers was taken to define MSI-high, as previously reported (20, 38). Eight CpG island methylator phenotype (CIMP)-specific promoters (*CACNA1G*, *CDKN2A*, *CRABP1*, *IGF2*, *MLH1*, *NEUROG1*, *RUNX3*, and *SOCS1*) were assessed using MethyLight assays on bisulfite-treated DNA, as described earlier (20, 38). CIMP-high status was assigned according to there being ≥6 of these 8 promoters that were methylated. Reciprocally, CIMP-low or negative was defined as having 0–5 methylated promoters, also as reported earlier (20). Methylation of long-interspersed nucleotide element-1 (LINE-1) was quantified by pyrosequencing of bisulfite-treated DNA, as previously described (20). PCR and pyrosequencing were carried out for *KRAS* (codons 12, 13, 61, and 146), *BRAF* (codon 600), and *PIK3CA* (exons 9 and 20) (20, 39).

### Statistical Analyses

The details of statistical analyses are described in **Supplementary Methods**. Briefly, all statistical analyses were performed using the SAS software (version 9.4, SAS Institute, Cary, NC). All P values were two-sided. We used the stringent two-sided α level of 0.005, accounting for multiple comparisons (40). Our primary hypothesis testing was conducted to assess the association of T-cell and macrophage densities (four ordinal categories) in intraepithelial and stromal regions with desmoplastic reaction (three categories), myxoid stroma (four-tiered scale), and keloid-like collagen bundles (four-tiered scale) in the multivariable ordinal logistic regression models. All the other hypotheses were tested as secondary analyses. The American Joint Committee on Cancer TNM staging criteria were used to evaluate the disease stage. Chi-squared test was used to assess the relationship between clinicopathological features and desmoplastic reaction. To adjust for selection bias due to the availability of tumor tissue samples, we applied the inverse probability weighting (IPW) method in logistic regression, Cox regression, and Kaplan–Meier analyses, utilizing covariate data of 4,465 incident colorectal cancer cases (26–28, 41). To control for potential confounders, we used a multivariable ordinal logistic regression model that calculated odds ratios (ORs) for one category increase in desmoplastic reaction categories in relation to T-cell and macrophage densities. P_trend_ was calculated by the linear trend across the ordinal categories of desmoplastic reaction, myxoid stroma, and keloid-like collagen bundles while adjusting for the same set of covariates. We also assessed a statistical interaction between T-cell/macrophage densities (four ordinal categories) and MSI status (high vs. non-high) for desmoplastic reaction (immature vs. intermediate vs. mature) using the Wald test for the cross-product term in multivariable logistic regression models. In the subgroup analysis of pT3 and pT4 cases, we assessed the association of T-cell and macrophage densities (four ordinal categories) in intraepithelial and stromal regions with desmoplastic reaction (three categories) in the multivariable ordinal logistic regression models.

In survival analyses, Kaplan–Meier method was used to estimate cumulative survival probabilities, and a linear trend in survival probability across ordinal categories of desmoplastic reaction, myxoid stroma, and keloid-like collagen bundles was determined using the log-rank test for trend. The inverse probability weighted multivariable Cox proportional hazard regression analyses were conducted for colorectal cancer-specific survival and overall survival according to desmoplastic reaction, myxoid stroma, and keloid-like collagen bundle grade. To control for potential confounders, we included the following covariates in the initial multivariable Cox regression model: age at diagnosis (continuous), sex (female vs. male), year of diagnosis (continuous), family history of colorectal cancer in any first-degree relative (present vs. absent), tumor location (proximal colon vs. distal colon vs. rectum), tumor differentiation (well-moderate vs. poor), American Joint Committee on Cancer (AJCC) disease stage (I-II vs. III-IV), MSI status (MSI-high vs. non-MSI-high), CIMP status (high vs. low/negative), LINE-1 methylation level (continuous), *KRAS* mutation (mutant vs. wild-type), *BRAF* mutation (mutant vs. wild-type), *PIK3CA* mutation (mutant vs. wild-type), tumor-infiltrating lymphocytes (negative/low vs. intermediate/high), intratumoral periglandular reaction (negative/low vs. intermediate/high), peritumoral lymphocytic reaction (negative/low vs. intermediate/high), Crohn’s-like lymphoid reaction negative/low vs. intermediate/high), intraepithelial CD3^+^CD8^+^CD45RO^+^ cell density (four ordinal categories), and stromal M1-like macrophage cell density (four ordinal categories). In addition, we assessed a statistical interaction between desmoplastic reaction/myxoid stroma/keloid-like collagen bundles (an ordinal variable) and T-cell/macrophage densities (a binary variable) for cancer-specific and overall survival using the Wald test for the cross-product term in multivariable-adjusted Cox regression models. In the subgroup analysis of AJCC disease stage I cases, we assessed colorectal cancer-specific survival and overall survival, according to the desmoplastic reaction, myxoid stroma, and keloid-like collagen bundle grade by using inverse probability weighted multivariable Cox proportional hazard regression models. A backward elimination was conducted with a threshold P of 0.05 to select variables for the final models in both logistic regression and Cox proportional hazard regression analyses.

## Results

Among the 4,465 patients, histopathological features of desmoplastic reaction were successfully assessed in 935 cases. Among those, we analyzed T-cell and macrophage densities and desmoplastic reaction in colorectal cancer tissue in 908 patients. Desmoplastic reaction at the invasive front was graded as mature, intermediate, and immature in 409 (45%), 230 (25%), and 269 (30%) cases, respectively. **Table 1**, **Supplementary Tables S1**, and **S2** summarize the clinical, pathological, and molecular characteristics. Immature desmoplastic reaction was associated with high pT, pN, and AJCC disease stage, poor tumor differentiation, lower intratumoral periglandular lymphocytic reaction, and lower peritumoral lymphocytic reaction (all P <0.0001).

**Table 1:** Clinical, pathological, and molecular characteristics of colorectal cancer cases according to desmoplastic reaction.

Characteristics^a^		Desmoplastic reaction			P value^b^
	Total No.	Mature	Intermediate	Immature	
	(n = 908)	(n = 409)	(n = 230)	(n = 269)	
Sex					0.0060
Female (NHS)	496 (55%)	200 (49%)	133 (58%)	163 (61%)	
Male (HPFS)	412 (45%)	209 (51%)	97 (42%)	106 (39%)	
Mean age ± SD (years)	69.1 ± 8.8	70.0 ± 8.7	68.7 ± 9.3	68.0 ± 8.5	0.014
Year of diagnosis					0.23
1995 or before	290 (32%)	125 (31%)	74 (32%)	91 (34%)	
1996–2000	298 (33%)	127 (32%)	73 (32%)	98 (36%)	
2001–2010	320 (35%)	157 (38%)	83 (36%)	80 (30%)	
Family history of colorectal cancer					0.38
in a first-degree relative				
Absent	709 (79%)	322 (79%)	186 (81%)	201 (76%)	
Present	191 (21%)	85 (21%)	43 (19%)	63 (24%)	
Tumor location					0.0017
Cecum	162 (18%)	63 (15%)	61 (27%)	38 (14%)	
Ascending to transverse colon	296 (33%)	127 (31%)	64 (28%)	105 (39%)	
Descending to sigmoid colon	267 (29%)	130 (32%)	63 (27%)	74 (28%)	
Rectum	179 (20%)	88 (22%)	41 (18%)	50 (19%)	
pT stage (depth of tumor invasion)					<0.0001
pT1 (submucosa)	65 (7.7%)	51 (13%)	13 (5.9%)	1 (0.4%)	
pT2 (muscularis propria)	172 (20%)	128 (34%)	34 (15%)	10 (4.1%)	
pT3 (subserosa)	560 (67%)	194 (51%)	157 (72%)	209 (86%)	
pT4 (serosa or other organs)	45 (5.3%)	6 (1.6%)	15 (6.9%)	24 (10%)	
pN stage					<0.0001
pN0	502 (61%)	301 (80%)	119 (57%)	82 (36%)	
pN1	201 (25%)	51 (13%)	68 (32%)	82 (36%)	
pN2	113 (14%)	26 (6.9%)	23 (11%)	64 (28%)	
AJCC disease stage					<0.0001
I	188 (22%)	148 (40%)	32 (14%)	8 (3.2%)	
II	281 (33%)	139 (37%)	83 (38%)	59 (23%)	
III	248 (29%)	69 (19%)	75 (34%)	104 (41%)	
IV	127 (15%)	16 (4.3%)	30 (14%)	81 (32%)	
Tumor differentiation					0.0004
Well to moderate	825 (91%)	385 (94%)	210 (91%)	230 (85%)	
Poor	82 (9.0%)	23 (6.0%)	20 (8.7%)	39 (15%)	
MSI status					0.18
Non-MSI-high	733 (83%)	329 (83%)	179 (80%)	225 (86%)	
MSI-high	150 (17%)	69 (17%)	45 (20%)	36 (14%)	
CIMP status					0.67
Low/negative	691 (82%)	316 (83%)	172 (80%)	203 (82%)	
High	154 (18%)	65 (17%)	43 (20%)	46 (18%)	
Mean LINE-1 methylation	62.5 ± 9.6	62.9 ± 9.6	62.6 ± 9.4	61.9 ± 9.9	0.48
level ± SD (%)				
*KRAS* mutation					0.91
Wild-type	518 (59%)	234 (59%)	132 (60%)	152 (58%)	
Mutant	363 (41%)	163 (41%)	89 (40%)	111 (42%)	
*BRAF* mutation					0.12
Wild-type	756 (85%)	349 (87%)	192 (85%)	215 (81%)	
Mutant	133 (15%)	51 (13%)	33 (15%)	49 (19%)	
*PIK3CA* mutation					0.59
Wild-type	697 (84%)	313 (82%)	179 (84%)	205 (85%)	
Mutant	137 (16%)	68 (18%)	33 (16%)	36 (15%)	
Tumor-infiltrating lymphocytes					0.014
Negative/low	651 (73%)	280 (69%)	158 (69%)	213 (81%)	
Intermediate	147 (16%)	75 (19%)	41 (18%)	31 (12%)	
High	99 (11%)	49 (12%)	30 (13%)	20 (7.6%)	
Intratumoral periglandular reaction					<0.0001
Negative/low	126 (14%)	34 (8.4%)	35 (15%)	57 (22%)	
Intermediate	664 (74%)	311 (77%)	162 (71%)	191 (72%)	
High	108 (12%)	59 (15%)	32 (14%)	17 (6.4%)	
Peritumoral lymphocytic reaction					<0.0001
Negative/low	145 (16%)	42 (11%)	39 (17%)	64 (24%)	
Intermediate	614 (69%)	287 (71%)	148 (65%)	179 (68%)	
High	137 (15%)	73 (18%)	42 (18%)	22 (8.3%)	
Crohn’s-like lymphoid reaction					0.11
Negative/low	574 (74%)	252 (74%)	138 (70%)	184 (79%)	
Intermediate	138 (18%)	58 (17%)	41 (21%)	39 (17%)	
High	59 (7.7%)	32 (9.3%)	17 (8.7%)	10 (4.3%)	

a Percentage indicates the proportion of patients with a specific clinical, pathological, or molecular characteristic among all patients or in the strata of desmoplastic reaction.

b To compare categorical data between the desmoplastic reaction classification, chi-squared test was performed. To compare continuous variables, an analysis of variance was performed.

AJCC, American Joint Committee on Cancer; CIMP, CpG island methylator phenotype; HPFS, Health Professionals Follow-up Study; LINE-1, long-interspersed nucleotide element-1; MSI, microsatellite instability; NHS, Nurses’ Health Study; SD, standard deviation.

In our primary hypothesis testing, we used both univariable and multivariable ordinal logistic regression analyses to assess the association of T-cell and macrophage densities in tumor intraepithelial and stromal regions with desmoplastic reaction, myxoid stroma, and keloid-like collagen bundles (**Tables 2**, **3**, **Supplementary Tables S3**, and **S4**). In multivariable analyses, higher intraepithelial densities of CD3^+^CD4^+^CD45RO^+^ cells, CD3^+^CD8^+^CD45RO^+^ cells, CD3^+^CD8^+^ cells, and CD3^+^ cells were inversely associated with immature desmoplastic reaction and myxoid stroma (all P_trend_ <0.001). Multivariable odds ratios (ORs) for the highest (C4) (vs. lowest C1 category) intraepithelial CD3^+^CD8^+^CD45RO^+^ cell density were 0.43 [95% confidence interval (CI) 0.29–0.62; P_trend_ <0.0001] for immature desmoplastic reaction, 0.33 (95% CI, 0.23–0.49; P_trend_ <0.0001) for myxoid stroma, and 0.44 (95% CI, 0.30–0.63; P_trend_ <0.0001) for keloid-like collagen bundles. In a subgroup of pT3 and pT4 cases (n = 605), 233 cases were classified into immature, 172 intermediate, and 200 mature. In multivariable analyses, higher intraepithelial densities of CD3^+^CD8^+^CD45RO^+^ cells were associated with immature desmoplastic reaction (P_trend_ = 0.0025) in pT3 and pT4 cases (**Supplementary Table S5**).

**Table 2:** Multivariable logistic regression analysis to assess the associations of T cell densities with desmoplastic reaction with IPW.

	Multivariable OR (95% CI)^a^		
	Immature desmoplastic reaction	Myxoid stroma	Keloid-like collagen bundles
CD3^+^ cell density			
Tumor intraepithelial region			
C1 (lowest)	1 (referent)	1 (referent)	1 (referent)
C2 (second)	0.68 (0.46–1.00)	0.61 (0.42–0.90)	0.83 (0.56–1.24)
C3 (third)	0.63 (0.42–0.92)	0.55 (0.37–0.81)	0.70 (0.47–1.04)
C4 (highest)	0.49 (0.33–0.73)	0.39 (0.26–0.59)	0.60 (0.41–0.90)
P_trend_ ^b^	0.0005	<0.0001	0.0079
Tumor stromal region			
C1 (lowest)	1 (referent)	1 (referent)	1 (referent)
C2 (second)	0.77 (0.52–1.14)	0.76 (0.52–1.12)	0.73 (0.49–1.07)
C3 (third)	0.56 (0.38–0.81)	0.52 (0.35–0.77)	0.64 (0.43–0.94)
C4 (highest)	0.55 (0.38–0.80)	0.47 (0.32–0.69)	0.67 (0.46–0.98)
P_trend_ ^b^	0.0006	<0.0001	0.032
CD3^+^CD4^+^ cell density			
Tumor intraepithelial region			
C1 (lowest)	1 (referent)	1 (referent)	1 (referent)
C2 (second)	0.68 (0.47–1.00)	0.63 (0.43–0.92)	0.70 (0.47–1.03)
C3 (third)	0.65 (0.45–0.95)	0.59 (0.41–0.85)	0.76 (0.52–1.13)
C4 (highest)	0.60 (0.41–0.88)	0.53 (0.36–0.78)	0.67 (0.47–0.96)
P_trend_ ^b^	0.0075	0.0011	0.047
Tumor stromal region			
C1 (lowest)	1 (referent)	1 (referent)	1 (referent)
C2 (second)	0.93 (0.63–1.37)	1.01 (0.69–1.47)	0.96 (0.65–1.43)
C3 (third)	0.79 (0.54–1.16)	0.76 (0.52–1.12)	0.96 (0.66–1.40)
C4 (highest)	0.64 (0.44–0.93)	0.55 (0.37–0.80)	0.75 (0.51–1.10)
P_trend_ ^b^	0.013	0.0007	0.16
CD3^+^CD8^+^ cell density			
Tumor intraepithelial region			
C1 (lowest)	1 (referent)	1 (referent)	1 (referent)
C2 (second)	0.78 (0.53–1.15)	0.89 (0.60–1.30)	0.71 (0.48–1.03)
C3 (third)	0.60 (0.42–0.86)	0.67 (0.47–0.95)	0.55 (0.38–0.79)
C4 (highest)	0.50 (0.35–0.73)	0.42 (0.29–0.62)	0.48 (0.33–0.69)
P_trend_ ^b^	<0.0001	<0.0001	<0.0001
Tumor stromal region			
C1 (lowest)	1 (referent)	1 (referent)	1 (referent)
C2 (second)	1.08 (0.75–1.56)	1.10 (0.76–1.60)	1.07 (0.73–1.55)
C3 (third)	0.85 (0.59–1.22)	0.89 (0.62–1.27)	0.85 (0.58–1.24)
C4 (highest)	0.74 (0.51–1.07)	0.61 (0.41–0.89)	0.71 (0.50–1.03)
P_trend_ ^b^	0.075	0.012	0.056
CD3^+^CD4^+^FOXP3^+^ cell density			
Tumor intraepithelial region			
C1 (lowest)	1 (referent)	1 (referent)	1 (referent)
C2 (second)	1.11 (0.72–1.72)	0.78 (0.49–1.25)	0.93 (0.58–1.49)
C3 (third)	0.78 (0.50–1.21)	0.89 (0.59–1.35)	0.73 (0.47–1.15)
C4 (highest)	0.90 (0.58–1.39)	0.72 (0.47–1.12)	0.79 (0.53–1.18)
P_trend_ ^b^	0.41	0.12	0.12
Tumor stromal region			
C1 (lowest)	1 (referent)	1 (referent)	1 (referent)
C2 (second)	0.70 (0.47–1.04)	0.74 (0.51–1.08)	0.73 (0.49–1.08)
C3 (third)	0.86 (0.59–1.26)	0.75 (0.51–1.10)	1.06 (0.72–1.57)
C4 (highest)	0.83 (0.57–1.22)	0.69 (0.47–1.02)	0.77 (0.54–1.11)
P_trend_ ^b^	0.26	0.030	0.29
CD3^+^CD4^+^CD45RO^+^ cell density			
Tumor intraepithelial region			
C1 (lowest)	1 (referent)	1 (referent)	1 (referent)
C2 (second)	0.69 (0.48–1.00)	0.69 (0.48–1.01)	0.76 (0.52–1.12)
C3 (third)	0.65 (0.45–0.94)	0.60 (0.42–0.86)	0.84 (0.57–1.23)
C4 (highest)	0.54 (0.37–0.77)	0.49 (0.34–0.72)	0.62 (0.43–0.89)
P_trend_ ^b^	0.0008	0.0001	0.020
Tumor stromal region			
C1 (lowest)	1 (referent)	1 (referent)	1 (referent)
C2 (second)	1.09 (0.74–1.61)	1.14 (0.78–1.67)	0.99 (0.67–1.47)
C3 (third)	0.78 (0.53–1.14)	0.77 (0.52–1.13)	0.99 (0.68–1.46)
C4 (highest)	0.64 (0.44–0.92)	0.54 (0.36–0.79)	0.73 (0.50–1.06)
P_trend_ ^b^	0.0054	0.0003	0.12
CD3^+^CD4^+^CD45RO^-^ cell density			
Tumor intraepithelial region			
C1 (lowest)	1 (referent)	1 (referent)	1 (referent)
C2 (second)	0.85 (0.57–1.26)	0.69 (0.46–1.03)	0.67 (0.44–1.04)
C3 (third)	0.99 (0.69–1.43)	0.82 (0.57–1.18)	0.98 (0.67–1.44)
C4 (highest)	0.96 (0.65–1.43)	0.80 (0.54–1.17)	0.93 (0.65–1.32)
P_trend_ ^b^	0.83	0.15	0.67
Tumor stromal region			
C1 (lowest)	1 (referent)	1 (referent)	1 (referent)
C2 (second)	0.71 (0.48–1.04)	0.90 (0.63–1.28)	0.73 (0.49–1.07)
C3 (third)	1.01 (0.70–1.47)	0.93 (0.63–1.36)	1.11 (0.75–1.62)
C4 (highest)	0.75 (0.52–1.08)	0.65 (0.45–0.94)	0.79 (0.56–1.11)
P_trend_ ^b^	0.27	0.042	0.42
CD3^+^CD8^+^CD45RO^+^ cell density			
Tumor intraepithelial region			
C1 (lowest)	1 (referent)	1 (referent)	1 (referent)
C2 (second)	0.53 (0.36–0.76)	0.59 (0.41–0.86)	0.56 (0.39–0.81)
C3 (third)	0.55 (0.38–0.80)	0.56 (0.39–0.80)	0.53 (0.36–0.78)
C4 (highest)	0.43 (0.29–0.62)	0.33 (0.23–0.49)	0.44 (0.30–0.63)
P_trend_ ^b^	<0.0001	<0.0001	<0.0001
Tumor stromal region			
C1 (lowest)	1 (referent)	1 (referent)	1 (referent)
C2 (second)	1.16 (0.81–1.66)	1.22 (0.84–1.77)	1.23 (0.84–1.80)
C3 (third)	0.85 (0.59–1.24)	0.88 (0.60–1.28)	0.83 (0.57–1.21)
C4 (highest)	0.67 (0.46–0.98)	0.54 (0.37–0.78)	0.71 (0.50–1.02)
P_trend_ ^b^	0.033	0.0025	0.049
CD3^+^CD8^+^CD45RO^-^ cell density			
Tumor intraepithelial region			
C1 (lowest)	1 (referent)	1 (referent)	1 (referent)
C2 (second)	1.12 (0.73–1.72)	1.14 (0.72–1.82)	0.97 (0.64–1.47)
C3 (third)	0.80 (0.52–1.22)	0.97 (0.66–1.43)	0.70 (0.47–1.04)
C4 (highest)	0.77 (0.51–1.16)	0.60 (0.39–0.92)	0.65 (0.43–0.97)
P_trend_ ^b^	0.15	0.056	0.014
Tumor stromal region			
C1 (lowest)	1 (referent)	1 (referent)	1 (referent)
C2 (second)	1.21 (0.82–1.79)	1.23 (0.84–1.82)	1.11 (0.75–1.65)
C3 (third)	0.88 (0.61–1.28)	0.85 (0.59–1.22)	0.93 (0.65–1.34)
C4 (highest)	1.12 (0.74–1.69)	0.92 (0.61–1.39)	0.84 (0.55–1.28)
P_trend_ ^b^	0.85	0.53	0.42

a The multivariable ordinal logistic regression model initially included age, sex, year of diagnosis, family history of colorectal cancer, tumor location, tumor grade, microsatellite instability, CpG island methylator phenotype, long-interspersed nucleotide element-1 methylation level, KRAS, BRAF, and PIK3CA mutations. A backward elimination with a threshold P of 0.05 was used to select variables for the final model.

b P_trend_ was calculated by the linear trend across the ordinal categories of the T-cell densities (C1–C4, as an ordinal predictor variable) in an ordinal logistic regression model for desmoplastic reaction (three categories), myxoid stroma (four categories), or keloid-like collagen bundles (four categories) (as an ordinal outcome variable). CI, confidence interval; OR, odds ratio; IPW, inverse probability weighting.

**Table 3:** Multivariable logistic regression analysis to assess the associations of macrophage densities with desmoplastic reaction with IPW.

	Multivariable OR (95% CI)^a^		
	Immature desmoplastic reaction^b^	Myxoid stroma	Keloid-like collagen bundles
**Overall macrophage density**			
** Tumor intraepithelial region**			
C1 (lowest)	1 (referent)	1 (referent)	1 (referent)
C2 (second)	0.81 (0.52–1.27)	0.65 (0.44–0.95)	0.84 (0.57–1.26)
C3 (third)	0.91 (0.57–1.45)	0.74 (0.50–1.10)	0.83 (0.55–1.26)
C4 (highest)	0.69 (0.43–1.11)	0.51 (0.35–0.76)	0.74 (0.50–1.10)
P_trend_ ^c^	0.20	0.0030	0.15
** Tumor stromal region**			
C1 (lowest)	1 (referent)	1 (referent)	1 (referent)
C2 (second)	0.73 (0.47–1.14)	0.83 (0.55–1.24)	0.98 (0.65–1.47)
C3 (third)	0.71 (0.45–1.11)	0.69 (0.47–1.03)	0.99 (0.66–1.48)
C4 (highest)	0.43 (0.27–0.69)	0.48 (0.32–0.71)	0.95 (0.64–1.41)
P_trend_ ^c^	0.0009	0.0002	0.82
**M1-like macrophage density**			
** Tumor intraepithelial region**			
C1 (lowest)	1 (referent)	1 (referent)	1 (referent)
C2 (second)	0.63 (0.39–1.00)	0.80 (0.55–1.18)	0.80 (0.53–1.19)
C3 (third)	0.97 (0.63–1.50)	0.85 (0.57–1.26)	0.89 (0.59–1.33)
C4 (highest)	0.64 (0.41–1.01)	0.64 (0.43–0.94)	0.66 (0.45–0.98)
P_trend_ ^c^	0.21	0.042	0.076
** Tumor stromal region**			
C1 (lowest)	1 (referent)	1 (referent)	1 (referent)
C2 (second)	0.66 (0.42–1.04)	0.73 (0.49–1.08)	0.83 (0.55–1.25)
C3 (third)	0.71 (0.46–1.11)	0.71 (0.48–1.05)	0.97 (0.65–1.44)
C4 (highest)	0.44 (0.28–0.70)	0.50 (0.34–0.74)	0.70 (0.47–1.03)
P_trend_ ^c^	0.0011	0.0007	0.14
**M2-like macrophage density**			
** Tumor intraepithelial region**			
C1 (lowest)	1 (referent)	1 (referent)	1 (referent)
C2 (second)	0.96 (0.61–1.51)	1.20 (0.81–1.78)	1.12 (0.74–1.69)
C3 (third)	0.94 (0.60–1.49)	0.93 (0.62–1.38)	1.02 (0.69–1.53)
C4 (highest)	0.83 (0.52–1.33)	0.82 (0.55–1.24)	1.00 (0.66–1.52)
P_trend_ ^c^	0.45	0.21	0.88
** Tumor stromal region**			
C1 (lowest)	1 (referent)	1 (referent)	1 (referent)
C2 (second)	1.09 (0.70–1.71)	1.05 (0.71–1.57)	1.20 (0.80–1.82)
C3 (third)	1.01 (0.64–1.60)	1.14 (0.77–1.68)	1.60 (1.09–2.37)
C4 (highest)	0.90 (0.57–1.44)	0.95 (0.65–1.39)	1.43 (0.96–2.12)
P_trend_ ^c^	0.60	0.90	0.032

a The multivariable ordinal logistic regression model initially included age, sex, year of diagnosis, family history of colorectal cancer, tumor location, tumor grade, microsatellite instability, CpG island methylator phenotype, long-interspersed nucleotide element-1 methylation level, KRAS, BRAF, and PIK3CA mutations. A backward elimination with a threshold P of 0.05 was used to select variables for the final model.

b To avoid violation of the proportional odds assumption, the binary categories were used for desmoplastic reaction (immature vs intermediate/mature).

c P_trend_ was calculated by the linear trend across the ordinal categories of the macrophage densities (C1–C4, as an ordinal predictor variable) in an ordinal logistic regression model for desmoplastic reaction (three categories), myxoid stroma (four categories), or keloid-like collagen bundles (four categories) (as an ordinal outcome variable).

CI, confidence interval; OR, odds ratio; IPW, inverse probability weighting.

Within the macrophage populations, higher tumor stromal M1-like macrophage densities were inversely associated with immature desmoplastic reaction [OR for the highest (vs. lowest) density category 0.44; 95% CI, 0.28–0.70; P_trend_ = 0.0011] and myxoid stroma [OR for the highest (vs. lowest) density category 0.50; 95% CI, 0.34–0.74; P_trend_ = 0.0007], while M2-like macrophage densities were not significantly associated with desmoplastic reaction, myxoid stroma, or keloid-like collagen bundles (P_trend_ >0.03, with the α level of 0.005). In the pT3 and pT4 case subgroup, higher overall tumor stromal macrophage densities were inversely associated with desmoplastic reaction (P_trend_ = 0.0036), and higher tumor stromal M1-like macrophage densities showed a tendency toward an inverse association with immature desmoplastic reaction (P_trend_ = 0.0056; with the α level of 0.005; **Supplementary Table S6**).

The inverse associations of intraepithelial CD3^+^CD8^+^CD45RO^+^ cell and stromal M1-like macrophage densities with immature desmoplastic reaction, myxoid stroma, and keloid-like collagen bundles did not significantly differ by tumor MSI status (all P_interaction_ >0.3; **Supplementary Table S7**).

**Figure 3:**
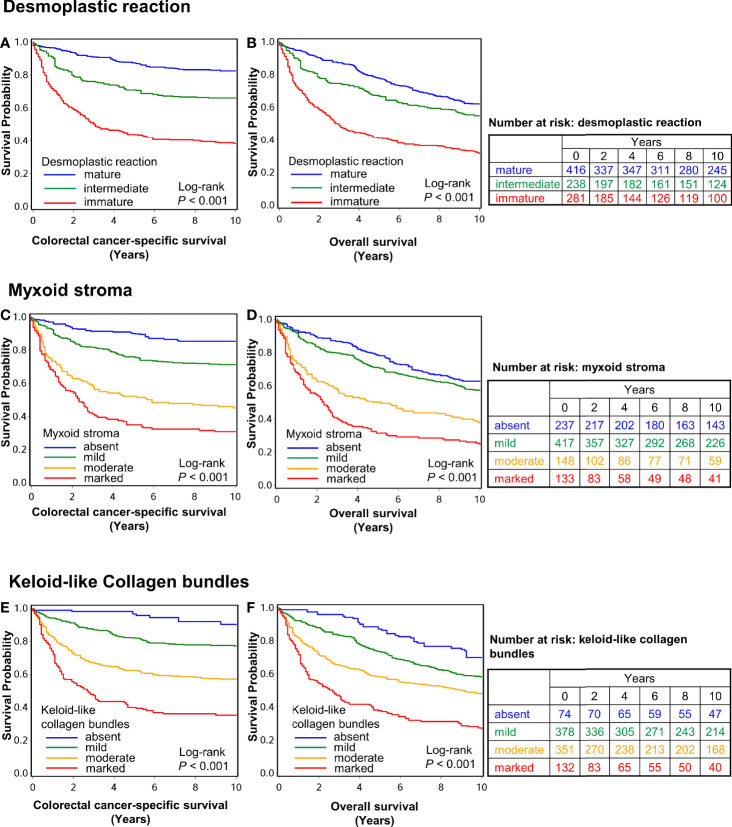
Inverse probability weighting-adjusted Kaplan–Meier survival curves of colorectal cancer-specific survival and overall survival. Panels **(A–F)** show survival data according to desmoplastic reaction **(A, B)**, myxoid stroma **(C, D)**, and keloid-like collagen bundles **(E, F)**. The P values were calculated using the weighted log-rank test for trend (two-sided). The tables show the number of patients who remained alive and at risk of death at each time point after the diagnosis of colorectal cancer.

**Figure 4:**
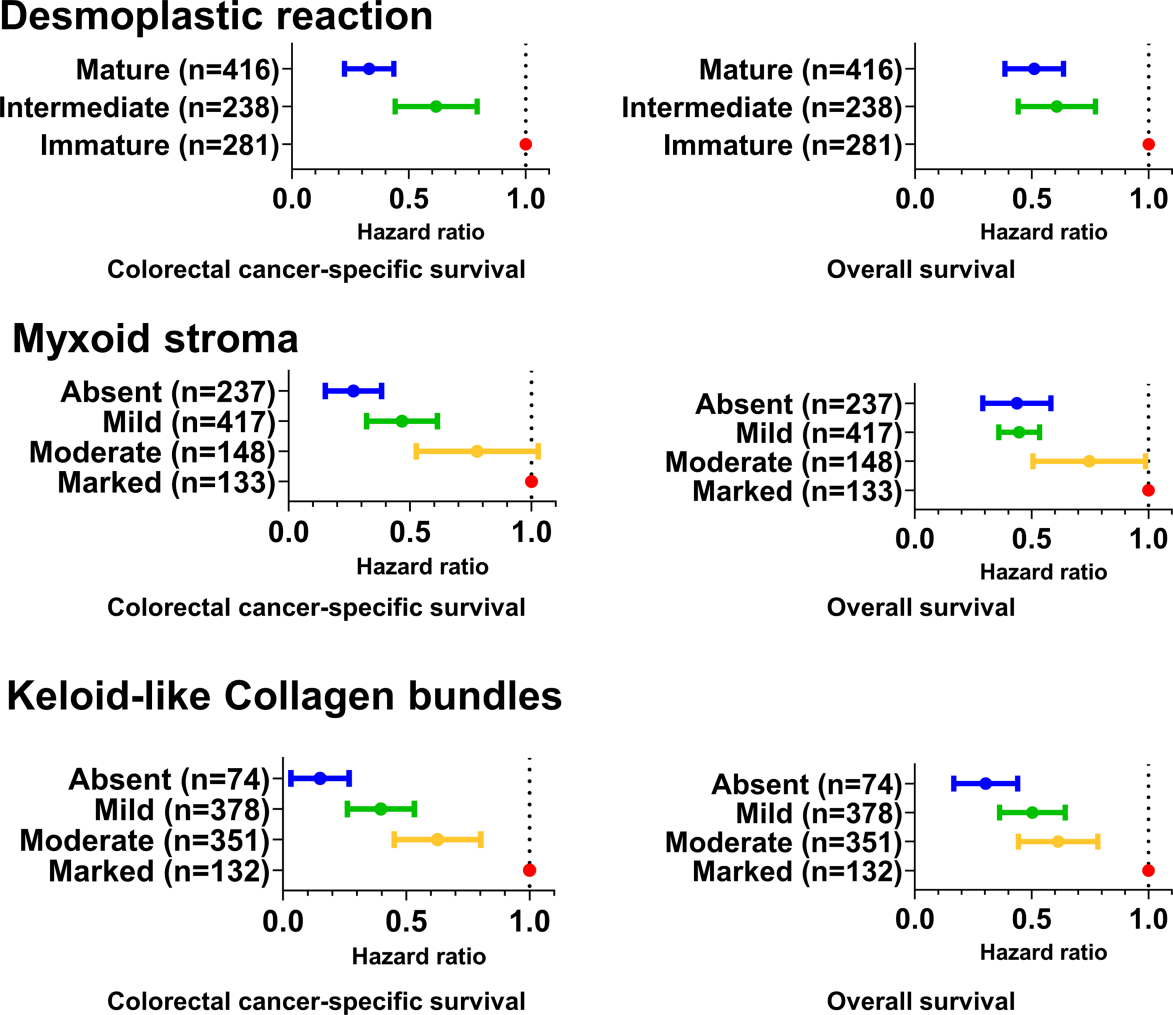
Forrest plot of multivariable Cox hazards regression analyses for cancer-specific and overall survival. The dots and vertical bars indicate the hazard ratios and 95% confidential intervals, respectively. The multivariable Cox regression models initially included sex, age, year of diagnosis, family history of colorectal cancer, tumor location, tumor grade, disease stage, microsatellite instability, CpG island methylator phenotype, long-interspersed nucleotide element-1 methylation level, *KRAS*, *BRAF*, and *PIK3CA* mutations, tumor-infiltrating lymphocytes, intratumoral periglandular reaction, peritumoral lymphocytic reaction, Crohn’s-like lymphoid reaction, intraepithelial CD3^+^CD8^+^CD45RO^+^ T-cell density, and stromal M1-like macrophage density. A backward elimination with a threshold P of 0.05 was used to select variables for the final models.

In the survival analyses, we evaluated the prognostic significance of the desmoplastic reaction, myxoid stroma, and keloid-like collagen bundles, as well as their statistical interactions with T-cell or macrophage densities, using 935 cases with available survival data. There were 663 all-cause deaths, including 300 colorectal cancer-specific deaths during the median follow-up time of 16.2 years (interquartile range, 12.8 to 20.2 years) for censored cases. Kaplan–Meier analysis showed that immature desmoplastic reaction, myxoid stroma, and keloid-like collagen bundles were associated with higher colorectal cancer-specific and overall mortality (all log-rank P <0.001) (**Figure 3**). Multivariable Cox regression models indicated that immature desmoplastic reaction, myxoid stroma, and keloid-like collagen bundles would be associated with worse prognosis. Due to the small numbers of deaths in some categories, we set patients with immature desmoplastic reaction, marked myxoid stroma, and marked keloid-like collagen bundles as references; multivariable-adjusted hazard ratios (HRs) were 0.32 (95% CI 0.23–0.44, P_trend_ <0.0001) for mature desmoplastic reaction, 0.25 (95% CI 0.16–0.39, P_trend_ <0.0001) for absent myxoid stroma, and 0.12 (95% CI 0.05–0.28, P_trend_ <0.0001) for absent keloid-like collagen bundles (**Table 4** and **Figure 4**). In the subgroup analysis of AJCC disease stage I cases, multivariable Cox regression models indicated that only myxoid stroma would be associated with worse prognosis, although the number of events was extremely small. Multivariable-adjusted HRs were 0.02 (95% CI 0.002–0.25, P_trend_ = 0.0006) for absent myxoid stroma (**Supplementary Table S8**). Given the associations of intraepithelial CD3^+^CD8^+^CD45RO^+^ cell and stromal M1-like macrophage densities with desmoplastic reaction, myxoid stroma, and/or keloid-like collagen bundles, we evaluated the prognostic impact of desmoplastic reaction, myxoid stroma, and keloid-like collagen bundles in strata of intraepithelial CD3^+^CD8^+^CD45RO^+^ cell and stromal M1-like macrophage densities. There were no statistically significant interactions between these immune cells and tumor stromal parameters in colorectal cancer-specific survival analyses (P_interaction_ >0.02, with the α level of 0.005) (**Supplementary Tables S9** and **S10** **)**. In the overall survival analyses, immature desmoplastic reaction and keloid-like collagen bundles appeared to have stronger survival association in tumors with low stromal M1-like macrophage densities (both P_interaction_ <0.0005) (**Supplementary Table S10**).

**Table 4:** Desmoplastic reaction and its components and patient survival with inverse probability weighting (IPW).

	No. of cases	Colorectal cancer-specific survival^a^			Overall survival^a^		
		No. of events	Univariable	Multivariable	No. of events	Univariable	Multivariable
			HR (95% CI)*	HR (95% CI)^b^		HR (95% CI)*	HR (95% CI)^b^
							
Desmoplastic reaction							
Immature	281	150	1 (referent)	1 (referent)	179	1 (referent)	1 (referent)
Intermediate	238	69	0.42 (0.31–0.57)	0.60 (0.45–0.80)	104	0.49 (0.38–0.64)	0.59 (0.45–0.78)
Mature	416	64	0.19 (0.14–0.26)	0.32 (0.23–0.44)	160	0.36 (0.29–0.46)	0.49 (0.38–0.63)
P_trend_ ^c^			<0.0001	<0.0001		<0.0001	<0.0001
							
Myxoid stroma							
C1 (marked)	133	79	1 (referent)	1 (referent)	91	1 (referent)	1 (referent)
C2 (moderate)	148	71	0.67 (0.48–0.93)	0.75 (0.54–1.04)	88	0.68 (0.49–0.93)	0.72 (0.52–1.00)
C3 (mild)	417	104	0.28 (0.21–0.38)	0.45 (0.33–0.62)	177	0.36 (0.27–0.47)	0.47 (0.35–0.52)
C4 (absent)	237	29	0.13 (0.08–0.20)	0.25 (0.16–0.39)	87	0.29 (0.21–0.40)	0.42 (0.30–0.59)
P_trend_ ^c^			<0.0001	<0.0001		<0.0001	<0.0001
							
Keloid-like collagen bundles							
C1 (marked)	132	73	1 (referent)	1 (referent)	90	1 (referent)	1 (referent)
C2 (moderate)	351	129	0.53 (0.40–0.72)	0.61 (0.46–0.81)	173	0.55 (0.41–0.72)	0.60 (0.45–0.79)
C3 (mild)	378	74	0.24 (0.17–0.33)	0.38 (0.27–0.54)	154	0.37 (0.28–0.48)	0.49 (0.37–0.65)
C4 (absent)	74	7	0.09 (0.04–0.19)	0.12 (0.05–0.28)	26	0.24 (0.15–0.38)	0.28 (0.18–0.45)
P_trend_ ^c^			<0.0001	<0.0001		<0.0001	<0.0001
							

a IPW was applied to reduce a bias due to the availability of tumor tissue after cancer diagnosis (see the Statistical Analysis subsection for details).

b The multivariable Cox regression model initially included sex, age, year of diagnosis, family history of colorectal cancer, tumor location, tumor grade, disease stage, microsatellite instability, CpG island methylator phenotype, long-interspersed nucleotide element-1 methylation level, KRAS, BRAF, and PIK3CA mutations, tumor-infiltrating lymphocytes, intratumoral periglandular reaction, peritumoral lymphocytic reaction, Crohn’s-like lymphoid reaction, intraepithelial CD3^+^CD8^+^CD45RO^+^ T cell density, and stroma M1-like macrophage density. A backward elimination with a threshold P of 0.05 was used to select variables for the final models.

c P_trend_ value was calculated by the linear trend across the ordinal categories of the desmoplastic reaction, myxoid stroma, and keloid-like collagen bundles in the IPW-adjusted Cox regression model.

CI, confidence interval; HR, hazard ratio; IPW, inverse probability weighting.

## Discussion

Previous studies have supported the clinical and pathological impact of desmoplastic reaction in colorectal cancer (6–13). However, no studies have evaluated the detailed relationships between desmoplastic reaction and immune cells in the colorectal carcinoma microenvironment, although an association between immature stroma and lower density of CD3 has been suggested (11). Hence, we tested the hypothesis that specific T-cell and macrophage populations in the colorectal cancer microenvironment might be associated with immature desmoplastic reaction. The current study is the largest to evaluate the detailed immune cell populations, including T cells and macrophage, in relation to desmoplastic reaction and its components. We found an inverse association of intraepithelial CD3^+^CD8^+^CD45RO^+^ memory cytotoxic T cells and stromal M1-like macrophages with immature desmoplastic reaction and its components (myxoid stroma and keloid-like collagen bundles). In the pT3 and pT4 case subgroup, a similar trend was observed as for the association between intraepithelial densities of CD3^+^CD8^+^CD45RO^+^ and stromal M1-like macrophages and desmoplastic reaction in multivariable analyses. Additionally, immature desmoplastic reaction and its components would be associated with worse colorectal cancer-specific survival. Considering there were only limited events from AJCC disease stage I cases, we could not assess the prognostic impact of desmoplastic reaction and its components accurately, although myxoid stroma showed statistically significant association with worse prognosis.

The immune response to cancer antigens manifests as an accumulation of chemokine-induced immune cells (42). However, the tumor microenvironment may also harbor numerous immunosuppressive factors, and it is crucial to understand their interactions with the antitumorigenic immune cells (42). Cancer-associated fibroblasts play a crucial role in the development of desmoplastic reaction and shape the tumor immune microenvironment by the expression of immunoregulatory molecules such as TGFB1 (transforming growth factor-beta) (4, 5). Cancer-associated fibroblasts may directly and indirectly impact antitumor immune reaction through recruitment of protumorigenic inflammatory cells, such as M2-like macrophages (43).

Cytotoxic CD8^+^ T cells are considered an important component of effective antitumor immune response. They may target cancer cells and infectious cells using PRF1 (perforin 1) from cytotoxic granules to penetrate the cell membrane and inject GZMB (granzyme B) to induce apoptosis (42). They also secrete interferon-gamma to induce macrophage phagocytic activity and to activate antigen-presenting cells. Furthermore, cytotoxic T cells express FASLG (Fas ligand, CD178), thereby inducing cancer cell apoptosis through its binding with FAS (Fas cell surface death receptor, CD95) (42). In a previous study evaluating the same two U.S. nationwide colorectal cancer cohorts as the present study, we found that high tumor intraepithelial densities of CD3^+^CD8^+^CD45RO^+^ memory cytotoxic T cells were inversely associated with tumor budding, suggesting that cytotoxic antitumor immunity suppresses tumor microinvasion (22). Our present study adds to these findings by revealing that the density of CD3^+^CD8^+^CD45RO^+^ memory cytotoxic T cells is lower in tumors with immature stroma, which is another poor prognostic histologic feature. This finding is in line with a previous study that showed that the number of CD3^+^ lymphocytes decreases according to loss of maturation of desmoplastic reaction (11), but the more exact subpopulations driving this association had previously been unclear. Our study was not able to assess the mechanisms underlying in this association. However, these findings may reflect the inability of these antitumor effector cells to penetrate the immature desmoplastic stroma and attack tumor cells, or alternatively, immunosuppressive factors present in the immature stroma that inhibit the cytotoxic antitumor immunity.

Macrophages may play roles in both antitumor defense and tumor development. The concept of macrophage polarization relates to the phenotypic state of macrophages at a given point in space and time (44). Macrophage polarization can be viewed as a spectrum from pro-inflammatory M1-like to anti-inflammatory M2-like populations, and there are no perfect markers for different polarization states (44). Under the stimulus of IFNG (interferon-gamma) and lipopolysaccharides, macrophages undergo M1 polarization and produce inflammatory cytokines such as TNF (tumor necrosis factor-alpha), thereby accelerating an inflammatory response (45), while M2-like macrophages produce anti-inflammatory cytokines, such as IL10 (interleukin-10) and TGFB1 (transforming growth factor-beta), inducing regulatory T cells and suppressing cytotoxic T-cell response (45). Our multiplex immunofluorescence assay contained two markers for M1-polarization and two markers for M2-polarization, enabling more accurate characterization of the macrophage polarization state than single-marker approaches. We have found the inverse relationship of immature desmoplastic reaction with stromal M1-like macrophages but not with M2-like macrophages. These findings suggest that M1-like macrophages may suppress maturing of desmoplastic reaction, while it is also possible that immature desmoplastic reaction may influence macrophages and their polarization.

This study has several limitations. First, we assessed T-cell and macrophage densities using tissue microarrays, and such tissue sampling may not be representative of the overall tumor. The tissue microarrays contained two to four tumor tissue cores from each tumor (34). We have confirmed that at least two tissue microarray cores can provide reasonably accurate immune cell density measurements when compared to more extensive sampling (unpublished data). Second, the data for desmoplastic reaction and its components were based on a pathologist’s visual evaluation. However, we evaluated the interobserver agreement between the two pathologists, which resulted in at least moderate agreements for desmoplastic reaction, myxoid stroma, and keloid-like collagen bundles (weighted kappa 0.52, 0.57, and 0.40, respectively). This level of agreement is in line with a previous report (median weighted kappa 0.58) (46). Third, we cannot exclude the possibility of reverse causation. Nonetheless, reverse causation may not be the sole explanation to the observed interaction between desmoplastic reaction and immune cell densities. Fourth, most of the subjects in this research were non-Hispanic whites. Our findings should be evaluated in different populations. Fifth, detailed data on cancer treatments were not available in our study. However, we adjusted multivariable models for clinical, tumor characteristics, and demographic features as we utilized the MPE database. In addition, the treatment decision was not made on the basis of desmoplastic reaction features because the desmoplastic reaction data were generally not available for treating physicians. Sixth, stromal maturity measurements were the only tumor stroma parameters utilized in this study. The associations between immune cell infiltrates and other established, prognostically relevant stromal parameters such as the tumor–stroma ratio represent important topics for further investigation.

Our study has several strengths. First, the colorectal cancer cases in this study were collected from a large number of hospitals throughout the U.S., which facilitates the generalizability of our results. Our study is also one of the largest so far to evaluate the prognostic value of desmoplastic reaction and its components in colorectal cancer. Although desmoplastic reaction and its components were strongly associated with T-cell and macrophage densities, these stromal parameters showed a prognostic value independent of immune cells and potential confounders. Further studies are warranted to assess whether myxoid stroma and keloid-like collagen bundles could be reproducibly evaluated for prognostication of colorectal cancer. Second, we utilize IPW methods in all survival analyses to specifically reduce the potential bias caused by the availability of tissue after colorectal cancer diagnosis (26–28, 41). Third, we utilized the comprehensive molecular pathological epidemiology dataset, which includes many potential confounding factors as well as detailed molecular data, which were utilized in the multivariable logistic regression model and Cox regression models (47–49). Fourth, we assessed immune cell densities by using multiplex immunofluorescence, which enabled simultaneous examination of multiple T-cell and macrophage markers and identification of specific T-cell and macrophage subsets that were not possible with conventional single-marker approaches.

Given the strong association between desmoplastic reaction and tumor-immune characteristics, it would be possible that our results may be useful for predicting the treatment effect of immunotherapy. Since our cohort lacks sufficient treatment data and is generally conducted before the use of immunotherapy for colorectal cancer, it is required to verify our hypothesis using a new cohort with sufficient treatment with an immune checkpoint inhibitor. If certain types of immune cells in tumor tissues, such as intraepithelial CD3^+^CD8^+^CD45RO^+^ cells or stromal M1-like macrophages, could be used to predict the efficacy of certain immunotherapies, they may be attractive as an innovative biomarker using colonoscopy biopsies or surgically resected tissues prior to treatment. Further studies are warranted to investigate the efficacy of specific immune cell subtypes as new biomarkers. In addition, it would be worthwhile to develop a more reliable classification of the stroma by using digital image analysis.

In conclusion, we have shown that intraepithelial CD3^+^CD8^+^CD45RO^+^ T cells and stromal M1-like macrophages are inversely associated with immature desmoplastic reaction and its components, supporting the role of those immune cells in the desmoplastic reaction maturity in the tumor immune microenvironment. Our study also suggests the potential role of the evaluation of the desmoplastic reaction and its components as prognostic markers.

## Data Availability Statement

The original contributions presented in the study are included in the article/**Supplementary Material**. Further inquiries can be directed to https://www.nurseshealthstudy.org/researchers/ and https://sites.sph.harvard.edu/hpfs/for-collaborators/.

## Ethics Statement

Informed consent was obtained from all study participants, and the study was approved by the institutional review boards of the Brigham and Women’s Hospital and Harvard T.H. Chan School of Public Health (Boston, MA), and those of participating registries as required. The patients/participants provided their written informed consent to participate in this study.

## Author Contributions

Study concept and design: NA, JV, and SO. Acquisition of tissue data: NA, JV, MZ, TU, KF, JB, RZ, KH, KA, ML, JK, TT, AC, JN, and SO. Statistical analysis: NA, TU, KF, and SO. Drafting of the manuscript: NA, JV, MZ, TU, and SO. Editing and critical revision for important intellectual contents: NA, JV, MZ, TU, KF, JB, RZ, KH, KA, ML, JK, TT, MS, XZ, KW, AC, JM, MG, JN, and SO. Obtained funding: MS, XZ, KW, AC, JM, MG, JN, and SO. All authors read and approved the final manuscript.

## Funding

This work was supported by the U.S. National Institutes of Health (NIH) grants (P01 CA87969; UM1 CA186107; P01 CA55075; UM1 CA167552; U01 CA167552; R35 CA253185 to AC; R35 CA197735 to SO; R01 CA151993 to SO; R01 CA248857 to SO); by Stand Up to Cancer Colorectal Cancer Dream Team Translational Research Grant (SU2C-AACR-DT22-17 to MG), administered by the American Association for Cancer Research, a scientific partner of SU2C; and by grants from the Project P Fund, The Friends of the Dana-Farber Cancer Institute, Bennett Family Fund, and the Entertainment Industry Foundation through National Colorectal Cancer Research Alliance and SU2C. XZ was supported by the American Cancer Society Research Scholar Grant (RSG NEC-130476). KF was supported by fellowship grants from the Uehara Memorial Foundation and Grant of The Clinical Research Promotion Foundation (2018). RZ was supported by a fellowship grant from Huazhong University of Science and Technology. KA and TU were supported by a grant from Overseas Research Fellowship (201860083 to KA; 201960541 to TU) from Japan Society for the Promotion of Science. KH was supported by fellowship grants from the Uehara Memorial Foundation and the Mitsukoshi Health and Welfare Foundation. AC is a Stuart and Suzanne Steele MGH Research Scholar. MG is supported by an ASCO Conquer Cancer Foundation Career Development Award. The content is solely the responsibility of the authors and does not necessarily represent the official views of NIH. The funders had no role in study design, data collection and analysis, decision to publish, or preparation of the manuscript.

## Conflict of Interest

JM has received institutional research funding from Boston Biomedical, has served as an advisor/consultant to COTA Healthcare, and served on a grant review panel for the National Comprehensive Cancer Network funded by Taiho Pharmaceutical. MG received research funding from Bristol-Myers Squibb, Merck, Servier, and Janssen. This study was not funded by any of these commercial entities. AC previously served as a consultant for Bayer Healthcare and Pfizer Inc. This study was not funded by Bayer Healthcare or Pfizer Inc.

The remaining authors declare that the research was conducted in the absence of any commercial or financial relationships that could be construed as a potential conflict of interest.

## Publisher’s Note

All claims expressed in this article are solely those of the authors and do not necessarily represent those of their affiliated organizations, or those of the publisher, the editors and the reviewers. Any product that may be evaluated in this article, or claim that may be made by its manufacturer, is not guaranteed or endorsed by the publisher.

## Abbreviations

AJCC, American Joint Committee on Cancer; CI, confidence interval; CIMP, CpG island methylator phenotype; FFPE, formalin-fixed paraffin-embedded; HPFS, Health Professionals Follow-up Study; LINE-1, long interspersed nucleotide element-1; MSI, microsatellite instability; NHS, Nurses’ Health Study; OR, odds ratio; PCR, polymerase chain reaction; SD, standard deviation; TNM, tumor, node and metastases.
