# Supplementary material for: Desmoplastic Reaction, Immune Cell Response, and Prognosis in Colorectal Cancer
Source: Front Immunol. 2022 Mar 22;13:840198. doi: 10.3389/fimmu.2022.840198 (PMC8980356; doi:10.3389/fimmu.2022.840198)
Supplement: Supplementary file 1 [file DataSheet_1.pdf]

Supplementary Material

Supplementary methods

Statistical analyses

All statistical analyses were performed using SAS software (version 9.4, SAS Institute, Cary, NC). All P values were two-sided. We used the stringent two-sided α level of 0.005, accounting for multiple comparisons (1). Our primary hypothesis testing was an assessment of the association of T cell and macrophage densities (four ordinal categories, C1-C4) in intraepithelial and stromal regions with desmoplastic reaction (Ueno criteria), myxoid stroma (four-tiered scale), and keloid-like collagen bundles (four-tiered scale) in the multivariable ordinal logistic analyses. All the other hypotheses were tested as secondary analyses. We used American Joint Committee on Cancer We used the chi-squared test to assess the associations of clinicopathological characteristics with desmoplastic reaction. We used the multivariable ordinal logistic regression model to estimate odds ratios (ORs) for one category increase in desmoplastic reaction categories in relation to T cell and macrophage densities. To control for potential confounders, the multivariable ordinal logistic regression model initially included the following confounders: age at diagnosis (continuous), sex (female vs. male), year of diagnosis (continuous), family history of colorectal cancer in any first-degree relative (present vs. absent), tumor location (proximal colon vs. distal colon vs. rectum), tumor differentiation (well to moderate vs. poor), microsatellite instability (MSI) status (MSI-high vs. non-MSI-high), CpG island methylator phenotype (CIMP) status (high vs. low/negative), long-interspersed nucleotide element-1 (LINE-1) methylation level (continuous), *KRAS* mutation (mutant vs. wild-type), *BRAF* mutation (mutant vs. wild-type), *PIK3CA* mutation (mutant vs. wild-type). A backward elimination was conducted with a threshold P of 0.05 to select variables for the final models. P_{trend} was calculated by the linear trend across the ordinal categories of desmoplastic reaction, myxoid stroma, and keloid-like collagen bundles with the same set of covariates. We also assessed a statistical interaction between T cell/macrophage densities (four ordinal categories) and MSI status (high vs. non-high) for desmoplastic reaction, myxoid stroma, and keloid-like collagen bundles using the Wald test for the cross-product term in multivariable logistic regression models. We estimated odds ratios (ORs) in the two strata of MSI status (MSI-high vs. non-MSI-high) using re-parameterization of the interaction term in one regression model (2). The proportional odds assumption was generally satisfied except for a model for desmoplastic reaction and macrophage. Therefore, we used a binary variable for desmoplastic reaction (immature vs. intermediate/mature) in this model.

In survival analyses, cumulative survival probabilities were estimated with the Kaplan-Meier method, and a linear trend in survival probability across ordinal categories of desmoplastic reaction, myxoid stroma, and keloid-like collagen bundles was determined using the log-rank test for trend. For analyses of colorectal cancer-specific mortality, deaths due to other causes were censored. Survival time was defined as the period from diagnosis of colorectal cancer to death or the end of follow-up, whichever came first. Multivariable Cox proportional hazard regression analyses were conducted for colorectal cancer-specific survival and overall survival according to desmoplastic reaction (immature vs. intermediate vs. mature), myxoid stroma (marked vs. moderate vs. mild vs. absent), and keloid-like collagen bundles grade (marked vs. moderate vs. mild vs. absent). A

backward elimination was conducted with a threshold P of 0.05 to select variables for the final models. P_{trend} was calculated by the linear trend across the ordinal categories of desmoplastic reaction, myxoid stroma, and keloid-like collagen bundles with the same set of covariates.

Additionally, we assessed a statistical interaction between desmoplastic reaction/keloid-like collagen bundles/myxoid stroma (an ordinal variable) and T cell/macrophage densities (a binary variable) for cancer-specific and overall survival using the Wald test for the cross-product term in multivariable Cox regression models. Intraepithelial $CD3^+CD8^+CD45RO^+$ T cell densities were additionally adjusted in analyses of an interaction between desmoplastic reaction/keloid-like collagen bundles/myxoid stroma and macrophage densities, and stromal M1-like macrophage densities were additionally adjusted in analyses of an interaction between desmoplastic reaction/keloid-like collagen bundles/myxoid stroma and T cell densities.

The proportionality of hazards assumption was assessed using a time varying covariate, which is an interaction term of survival time and desmoplastic reaction. For cancer-specific survival, the proportionality of hazards assumption was generally satisfied ($P > 0.07$). For overall survival, the Schoenfeld residual plots supported the proportionality of hazards during the follow-up period up to 10 years (data not shown), and thus we used Cox regression models limiting the follow-up period to 10 years in analyses of overall survival.

Cases with missing data [family history of colorectal cancer in a first-degree relative (0.9%), tumor location (0.4%), MSI status (2.8%), CIMP status (6.9%), *KRAS* (3.0%), *BRAF* (2.1%), and *PIK3CA* mutation (8.2%), tumor-infiltrating lymphocytes (1.2%), intratumoral periglandular reaction (1.1%), peritumoral lymphocytic reaction (1.3%)] were included in the majority category of a given categorical covariate to limit the degrees of freedom of the models. For cases with missing data regarding LINE-1 methylation (2.6%), we imputed the mean value and assigned a separate indicator variable. For cases with missing data on Crohn's-like lymphoid reaction (15%), we assigned a separate indicator variable. We confirmed that excluding the cases with missing information in any of the covariates did not substantially alter results (data not shown).

To adjust for selection bias due to the availability of tumor tissue samples, we applied the inverse probability weighting (IPW) method in logistic regression, Cox regression, and Kaplan-Meier analyses, utilizing covariate data of 4,465 incident colorectal cancer cases (2-5). We generated a multivariable logistic regression model to estimate the probability of the tumor tissue availability (6). Each patient with available tissue data was weighted by the inverse of the probability. Weights greater than the 95th percentile were truncated and set to the value of the 95th percentile to reduce outlier effects (3). We confirmed that results without weighted truncation did not change substantially (data not shown).

References

1. Benjamin DJ, Berger JO, Johannesson M, Nosek BA, Wagenmakers EJ, Berk R, et al. Redefine statistical significance. Nat Hum Behav. (2018);2:6-10. doi:10.1038/s41562-017-0189-z.
2. Hamada T, Cao Y, Qian ZR, Masugi Y, Nowak JA, Yang J, et al. Aspirin Use and Colorectal Cancer Survival According to Tumor CD274 (Programmed Cell Death 1 Ligand 1) Expression Status. J Clin Oncol. (2017);35:1836-1844. doi:10.1200/jco.2016.70.7547.
3. Seaman SR, White IR. Review of inverse probability weighting for dealing with missing data. Stat Methods Med Res. (2013);22:278-95. doi:10.1177/0962280210395740.

4. Xie J, Liu C. Adjusted Kaplan-Meier estimator and log-rank test with inverse probability of treatment weighting for survival data. Stat Med. (2005);24:3089-110. doi:10.1002/sim.2174.

5. Liu L, Nevo D, Nishihara R, Cao Y, Song M, Twombly TS, et al. Utility of inverse probability weighting in molecular pathological epidemiology. Eur J Epidemiol. (2018);33:381-392. doi:10.1007/s10654-017-0346-8.

6. Kosumi K, Hamada T, Koh H, Borowsky J, Bullman S, Twombly TS, et al. The Amount of Bifidobacterium Genus in Colorectal Carcinoma Tissue in Relation to Tumor Characteristics and Clinical Outcome. Am J Pathol. (2018);188:2839-2852. doi:10.1016/j.ajpath.2018.08.015.

Supplementary Tables

Supplementary Table S1. Clinical, pathological, and molecular characteristics of colorectal cancer cases according to myxoid stroma

Characteristics[a]	Total No. (n = 908)	Myxoid stroma Absent (n = 230)	Mild (n = 409)	Moderate (n = 137)	Marked (n = 132)	P value[b]
Sex						0.042
Female (NHS)	496 (55%)	110 (48%)	223 (55%)	84 (61%)	79 (60%)	
Male (HPFS)	412 (45%)	120 (52%)	186 (45%)	53 (39%)	53 (40%)	
Mean age ± SD (years)	69.1 ± 8.8	70.3 ± 8.8	69.1 ± 9.0	67.8 ± 8.3	68.2 ± 8.6	0.036
Year of diagnosis						0.30
1995 or before	290 (32%)	66 (29%)	133 (32%)	50 (37%)	41 (31%)	
1996-2000	298 (33%)	75 (32%)	125 (31%)	47 (34%)	51 (39%)	
2001-2010	320 (35%)	89 (39%)	151 (37%)	40 (29%)	40 (30%)	
Family history of colorectal cancer in a first-degree relative						0.26
Absent	709 (79%)	185 (81%)	323 (79%)	98 (73%)	103 (80%)	
Present	191 (21%)	43 (19%)	85 (21%)	37 (27%)	26 (20%)	
Tumor location						0.040
Cecum	162 (18%)	36 (16%)	88 (21%)	18 (13%)	20 (15%)	
Ascending to transverse colon	296 (33%)	62 (27%)	129 (32%)	56 (42%)	49 (37%)	
Descending to sigmoid colon	267 (29%)	75 (33%)	118 (29%)	39 (29%)	35 (27%)	
Rectum	179 (20%)	56 (24%)	73 (18%)	22 (16%)	28 (21%)	
pT stage (depth of tumor invasion)						<0.0001
pT1 (submucosa)	65 (7.7%)	39 (18%)	25 (6.5%)	1 (0.8%)	0 (0%)	
pT2 (muscularis propria)	172 (20%)	86 (41%)	76 (20%)	7 (5.6%)	3 (2.5%)	
pT3 (subserosa)	560 (67%)	86 (41%)	265 (69%)	110 (87%)	99 (84%)	
pT4 (serosa or other organs)	45 (5.3%)	0 (0%)	21 (5.4%)	8 (6.4%)	16 (14%)	
pN stage						<0.0001
pN0	502 (61%)	170 (81%)	250 (66%)	49 (42%)	33 (30%)	
pN1	201 (25%)	30 (14%)	89 (23%)	44 (37%)	38 (35%)	
pN2	113 (14%)	9 (4.3%)	40 (11%)	25 (21%)	39 (35%)	
AJCC disease stage						<0.0001
I	188 (22%)	103 (50%)	77 (20%)	6 (4.7%)	2 (1.6%)	
II	281 (33%)	59 (29%)	163 (42%)	35 (27%)	24 (20%)	
III	248 (29%)	35 (17%)	109 (28%)	53 (41%)	51 (41%)	
IV	127 (15%)	10 (4.8%)	36 (9.4%)	35 (27%)	46 (37%)	
Tumor differentiation						0.0012
Well to moderate	825 (91%)	215 (94%)	380 (93%)	114 (83%)	116 (88%)	
Poor	82 (9.0%)	14 (6.1%)	29 (7.1%)	23 (17%)	16 (12%)	
MSI status						0.44
Non-MSI-high	733 (83%)	183 (82%)	325 (82%)	114 (86%)	111 (87%)	
MSI-high	150 (17%)	41 (18%)	73 (18%)	19 (14%)	17 (13%)	

CIMP status						0.99
Low/negative	691 (82%)	172 (82%)	316 (82%)	105 (82%)	98 (81%)	
High	154 (18%)	38 (18%)	70 (18%)	23 (18%)	23 (19%)	
Mean LINE-1 methylation level ± SD (%)	62.5 ± 9.6	62.7 ± 9.4	62.8 ± 9.6	62.3 ± 10.4	61.6 ± 9.3	0.63
KRAS mutation						0.037
Wild-type	518 (59%)	149 (67%)	217 (55%)	77 (58%)	75 (58%)	
Mutant	363 (41%)	74 (33%)	178 (45%)	56 (42%)	55 (42%)	
BRAF mutation						0.25
Wild-type	756 (85%)	197 (88%)	344 (86%)	108 (81%)	107 (82%)	
Mutant	133 (15%)	28 (12%)	56 (14%)	25 (19%)	24 (18%)	
PIK3CA mutation						0.13
Wild-type	697 (84%)	185 (85%)	307 (82%)	99 (80%)	106 (90%)	
Mutant	137 (16%)	32 (15%)	69 (18%)	24 (20%)	12 (10%)	
Tumor-infiltrating lymphocytes						0.0007
Negative/low	651 (73%)	150 (67%)	288 (71%)	100 (74%)	113 (88%)	
Intermediate	147 (16%)	46 (20%)	70 (17%)	25 (19%)	6 (4.7%)	
High	99 (11%)	30 (13%)	49 (12%)	10 (7.4%)	10 (7.8%)	
Intratumoral periglandular reaction						<0.0001
Negative/low	126 (14%)	19 (8.4%)	50 (12%)	30 (22%)	27 (21%)	
Intermediate	664 (74%)	168 (74%)	305 (75%)	97 (72%)	94 (72%)	
High	108 (12%)	39 (17%)	52 (13%)	8 (5.9%)	9 (6.9%)	
Peritumoral lymphocytic reaction						<0.0001
Negative/low	145 (16%)	21 (9.3%)	60 (15%)	31 (23%)	33 (25%)	
Intermediate	614 (69%)	156 (69%)	279 (69%)	94 (70%)	85 (65%)	
High	137 (15%)	49 (22%)	66 (16%)	10 (7.4%)	12 (9.2%)	
Crohn's-like lymphoid reaction						0.22
Negative/low	574 (74%)	136 (73%)	254 (72%)	90 (78%)	94 (80%)	
Intermediate	138 (18%)	30 (16%)	69 (20%)	21 (18%)	18 (15%)	
High	59 (7.7%)	20 (11%)	29 (8.2%)	5 (4.3%)	5 (4.3%)	

[a] Percentage indicates the proportion of patients with a specific clinical, pathological, or molecular characteristic among all patients or in the strata of myxoid stroma.
[b] To compare categorical data between the myxoid stroma classification, chi-square test was performed. To compare continuous variables, an analysis of variance was performed.
Abbreviations: AJCC, American Joint Committee on Cancer; CIMP, CpG island methylator phenotype; HPFS, Health Professionals Follow-up Study; LINE-1, long-interspersed nucleotide element-1; MSI, microsatellite instability; NHS, Nurses' Health Study; SD, standard deviation.

Supplementary Table S2. Clinical, pathological, and molecular characteristics of colorectal cancer cases according to keloid-like collagen bundles

Characteristics[a]	Total No. (n = 908)	Keloid-like collagen bundles Absent (n = 73)	Mild (n = 370)	Moderate (n = 337)	Marked (n = 128)	P value[b]
Sex						0.044
Female (NHS)	496 (55%)	35 (48%)	187 (51%)	203 (60%)	71 (55%)	
Male (HPFS)	412 (45%)	38 (52%)	183 (49%)	134 (40%)	57 (45%)	
Mean age ± SD (years)	69.1 ± 8.8	69.6 ± 9.9	69.7 ± 8.4	68.2 ± 8.5	69.1 ± 9.9	0.14
Year of diagnosis						0.26
1995 or before	290 (32%)	29 (40%)	107 (29%)	109 (32%)	45 (35%)	
1996-2000	298 (33%)	16 (22%)	126 (34%)	118 (35%)	38 (30%)	
2001-2010	320 (35%)	28 (38%)	137 (37%)	110 (23%)	45 (35%)	
Family history of colorectal cancer in a first-degree relative						0.98
Absent	709 (79%)	58 (81%)	288 (78%)	264 (79%)	99 (79%)	
Present	191 (21%)	14 (19%)	80 (22%)	71 (21%)	26 (21%)	
Tumor location						0.020
Cecum	162 (18%)	10 (14%)	58 (16%)	69 (21%)	25 (20%)	
Ascending to transverse colon	296 (33%)	14 (19%)	125 (34%)	115 (34%)	42 (33%)	
Descending to sigmoid colon	267 (29%)	24 (33%)	119 (32%)	92 (27%)	32 (25%)	
Rectum	179 (20%)	25 (34%)	67 (18%)	59 (18%)	28 (22%)	
pT stage (depth of tumor invasion)						<0.0001
pT1 (submucosa)	65 (7.7%)	10 (16%)	42 (12%)	12 (3.8%)	1 (0.9%)	
pT2 (muscularis propria)	172 (20%)	30 (47%)	101 (29%)	31 (10%)	10 (8.5%)	
pT3 (subserosa)	560 (67%)	24 (37%)	197 (57%)	243 (78%)	96 (81%)	
pT4 (serosa or other organs)	45 (5.3%)	0 (0%)	7 (2.0%)	27 (8.6%)	11 (9.3%)	
pN stage						<0.0001
pN0	502 (61%)	51 (84%)	266 (77%)	142 (48%)	43 (39%)	
pN1	201 (25%)	7 (11%)	53 (15%)	103 (35%)	38 (34%)	
pN2	113 (14%)	3 (4.9%)	28 (8.1%)	52 (17%)	30 (27%)	
AJCC disease stage						<0.0001
I	188 (22%)	32 (55%)	118 (34%)	31 (10%)	7 (5.7%)	
II	281 (33%)	16 (28%)	134 (39%)	102 (32%)	29 (24%)	
III	248 (29%)	10 (17%)	73 (21%)	117 (37%)	48 (39%)	
IV	127 (15%)	0 (0%)	22 (6.3%)	67 (21%)	38 (31%)	
Tumor differentiation						0.0007
Well to moderate	825 (91%)	72 (100%)	346 (94%)	294 (87%)	113 (88%)	
Poor	82 (9.0%)	0 (6.1%)	24 (6.5%)	43 (13%)	15 (12%)	
MSI status						0.47
Non-MSI-high	733 (83%)	62 (87%)	295 (82%)	268 (82%)	108 (86%)	
MSI-high	150 (17%)	9 (13%)	64 (18%)	60 (18%)	17 (14%)	

CIMP status						0.37
Low/negative	691 (82%)	61 (88%)	282 (82%)	252 (80%)	96 (83%)	
High	154 (18%)	8 (12%)	63 (18%)	64 (20%)	19 (17%)	
Mean LINE-1 methylation level ± SD (%)	62.5 ± 9.6	60.9 ± 10.9	62.6 ± 9.6	62.9 ± 9.5	62.0 ± 9.3	0.40
KRAS mutation						0.31
Wild-type	518 (59%)	42 (58%)	209 (58%)	201 (62%)	66 (52%)	
Mutant	363 (41%)	30 (42%)	150 (42%)	123 (38%)	60 (48%)	
BRAF mutation						0.053
Wild-type	756 (85%)	67 (93%)	313 (86%)	267 (81%)	109 (86%)	
Mutant	133 (15%)	5 (6.9%)	49 (14%)	61 (19%)	18 (14%)	
PIK3CA mutation						0.010
Wild-type	697 (84%)	62 (90%)	276 (81%)	265 (88%)	94 (77%)	
Mutant	137 (16%)	7 (10%)	65 (19%)	37 (12%)	28 (23%)	
Tumor-infiltrating lymphocytes						0.22
Negative/low	651 (73%)	48 (69%)	257 (70%)	245 (73%)	101 (81%)	
Intermediate	147 (16%)	16 (23%)	63 (17%)	53 (16%)	15 (12%)	
High	99 (11%)	6 (8.6%)	47 (13%)	37 (11%)	9 (7.2%)	
Intratumoral periglandular reaction						0.018
Negative/low	126 (14%)	6 (8.6%)	36 (9.8%)	59 (18%)	25 (20%)	
Intermediate	664 (74%)	56 (80%)	280 (76%)	241 (71%)	87 (70%)	
High	108 (12%)	8 (11%)	51 (14%)	36 (11%)	13 (10%)	
Peritumoral lymphocytic reaction						0.017
Negative/low	145 (16%)	9 (13%)	41 (11%)	66 (20%)	29 (23%)	
Intermediate	614 (69%)	50 (71%)	261 (72%)	225 (67%)	78 (63%)	
High	137 (15%)	11 (16%)	63 (17%)	45 (13%)	18 (14%)	
Crohn's-like lymphoid reaction						0.69
Negative/low	574 (74%)	47 (81%)	223 (72%)	219 (75%)	85 (76%)	
Intermediate	138 (18%)	6 (10%)	59 (19%)	54 (18%)	19 (17%)	
High	59 (7.7%)	5 (8.6%)	27 (8.7%)	19 (6.5%)	8 (7.1%)	

[a] Percentage indicates the proportion of patients with a specific clinical, pathological, or molecular characteristic among all patients or in the strata of keloid-like collagen bundles.
[b] To compare categorical data between the keloid-like collagen bundles classification, chi-square test was performed. To compare continuous variables, an analysis of variance was performed.
Abbreviations: AJCC, American Joint Committee on Cancer; CIMP, CpG island methylator phenotype; HPFS, Health Professionals Follow-up Study; LINE-1, long-interspersed nucleotide element-1; MSI, microsatellite instability; NHS, Nurses' Health Study; SD, standard deviation.

Supplementary Table S3. Univariable logistic regression analysis to assess the associations of T-cell with desmoplastic reaction with IPW

	Univariable OR (95% CI)		
	Immature desmoplastic reaction	Myxoid stroma	Keloid-like collagen bundles
**CD3$^+$ cell density**			
**Tumor intraepithelial region**			
C1 (lowest)	1 (referent)	1 (referent)	1 (referent)
C2 (second)	0.70 (0.47-1.02)	0.62 (0.42-0.92)	0.85 (0.57-1.27)
C3 (third)	0.60 (0.41-0.88)	0.55 (0.38-0.81)	0.69 (0.47-1.03)
C4 (highest)	0.52 (0.35-0.77)	0.41 (0.27-0.61)	0.66 (0.45-0.97)
P_{trend}[a]	0.0007	<0.0001	0.021
**Tumor stromal region**			
C1 (lowest)	1 (referent)	1 (referent)	1 (referent)
C2 (second)	0.77 (0.52-1.14)	0.79 (0.54-1.16)	0.74 (0.50-1.09)
C3 (third)	0.56 (0.38-0.81)	0.52 (0.35-0.77)	0.63 (0.43-0.94)
C4 (highest)	0.60 (0.41-0.87)	0.50 (0.34-0.73)	0.71 (0.49-1.03)
P_{trend}[a]	0.0024	<0.0001	0.059
**CD3$^+$CD4$^+$ cell density**			
**Tumor intraepithelial region**			
C1 (lowest)	1 (referent)	1 (referent)	1 (referent)
C2 (second)	0.70 (0.48-1.02)	0.68 (0.46-0.99)	0.73 (0.49-1.07)
C3 (third)	0.64 (0.44-0.93)	0.59 (0.41-0.85)	0.76 (0.52-1.13)
C4 (highest)	0.64 (0.44-0.93)	0.55 (0.37-0.80)	0.71 (0.50-1.02)
P_{trend}[a]	0.015	0.0012	0.085
**Tumor stromal region**			
C1 (lowest)	1 (referent)	1 (referent)	1 (referent)
C2 (second)	0.95 (0.65-1.40)	1.03 (0.70-1.50)	0.98 (0.66-1.45)
C3 (third)	0.77 (0.53-1.13)	0.73 (0.50-1.08)	0.94 (0.64-1.38)
C4 (highest)	0.66 (0.46-0.96)	0.56 (0.38-0.82)	0.78 (0.53-1.14)
P_{trend}[a]	0.017	0.0007	0.20
**CD3$^+$CD8$^+$ cell density**			
**Tumor intraepithelial region**			
C1 (lowest)	1 (referent)	1 (referent)	1 (referent)
C2 (second)	0.77 (0.53-1.13)	0.89 (0.61-1.29)	0.69 (0.48-1.01)
C3 (third)	0.59 (0.41-0.85)	0.66 (0.47-0.94)	0.55 (0.38-0.80)
C4 (highest)	0.52 (0.36-0.75)	0.42 (0.29-0.62)	0.50 (0.35-0.72)
P_{trend}[a]	<0.0001	<0.0001	<0.0001
**Tumor stromal region**			
C1 (lowest)	1 (referent)	1 (referent)	1 (referent)
C2 (second)	1.02 (0.71-1.47)	1.06 (0.74-1.53)	1.02 (0.70-1.48)
C3 (third)	0.80 (0.55-1.15)	0.85 (0.59-1.22)	0.81 (0.56-1.19)
C4 (highest)	0.73 (0.50-1.06)	0.60 (0.41-0.87)	0.72 (0.50-1.04)
P_{trend}[a]	0.058	0.0077	0.057
**CD3$^+$CD4$^+$FOXP3$^+$ cell density**			
**Tumor intraepithelial region**			
C1 (lowest)	1 (referent)	1 (referent)	1 (referent)
C2 (second)	1.19 (0.77-1.84)	0.83 (0.52-1.34)	1.00 (0.62-1.60)
C3 (third)	0.76 (0.49-1.18)	0.84 (0.56-1.26)	0.71 (0.45-1.11)
C4 (highest)	0.87 (0.57-1.34)	0.70 (0.46-1.08)	0.81 (0.54-1.21)
P_{trend}[a]	0.34	0.077	0.13
**Tumor stromal region**			
C1 (lowest)	1 (referent)	1 (referent)	1 (referent)
C2 (second)	0.68 (0.47-1.00)	0.74 (0.51-1.07)	0.75 (0.51-1.11)
C3 (third)	0.80 (0.55-1.16)	0.69 (0.47-1.01)	1.03 (0.70-1.52)
C4 (highest)	0.81 (0.55-1.18)	0.67 (0.46-0.98)	0.77 (0.53-1.10)
P_{trend}[a]	0.15	0.013	0.26

**$CD3^+CD4^+CD45RO^+$ cell density**			
**Tumor intraepithelial region**			
C1 (lowest)	1 (referent)	1 (referent)	1 (referent)
C2 (second)	0.68 (0.47-0.99)	0.70 (0.48-1.02)	0.76 (0.52-1.12)
C3 (third)	0.62 (0.43-0.90)	0.59 (0.41-0.84)	0.81 (0.55-1.18)
C4 (highest)	0.57 (0.39-0.82)	0.51 (0.35-0.74)	0.66 (0.46-0.95)
P_{trend}[a]	0.0015	0.0002	0.036
**Tumor stromal region**			
C1 (lowest)	1 (referent)	1 (referent)	1 (referent)
C2 (second)	1.11 (0.76-1.64)	1.14 (0.78-1.67)	1.05 (0.71-1.55)
C3 (third)	0.76 (0.52-1.12)	0.75 (0.51-1.10)	0.95 (0.65-1.40)
C4 (highest)	0.66 (0.45-0.95)	0.54 (0.37-0.79)	0.77 (0.53-1.12)
P_{trend}[a]	0.0073	0.0003	0.16
**$CD3^+CD4^+CD45RO^-$ cell density**			
**Tumor intraepithelial region**			
C1 (lowest)	1 (referent)	1 (referent)	1 (referent)
C2 (second)	0.89 (0.60-1.32)	0.75 (0.50-1.12)	0.70 (0.46-1.08)
C3 (third)	0.99 (0.69-1.44)	0.84 (0.59-1.21)	1.00 (0.68-1.47)
C4 (highest)	1.01 (0.69-1.50)	0.78 (0.53-1.15)	0.97 (0.68-1.38)
P_{trend}[a]	0.99	0.14	0.85
**Tumor stromal region**			
C1 (lowest)	1 (referent)	1 (referent)	1 (referent)
C2 (second)	0.74 (0.51-1.08)	0.93 (0.66-1.33)	0.73 (0.50-1.08)
C3 (third)	0.99 (0.68-1.43)	0.89 (0.61-1.30)	1.10 (0.75-1.61)
C4 (highest)	0.81 (0.56-1.15)	0.66 (0.46-0.95)	0.80 (0.57-1.13)
P_{trend}[a]	0.37	0.038	0.48
**$CD3^+CD8^+CD45RO^+$ cell density**			
**Tumor intraepithelial region**			
C1 (lowest)	1 (referent)	1 (referent)	1 (referent)
C2 (second)	0.53 (0.36-0.78)	0.61 (0.42-0.87)	0.57 (0.39-0.82)
C3 (third)	0.54 (0.38-0.79)	0.58 (0.41-0.82)	0.53 (0.36-0.78)
C4 (highest)	0.45 (0.31-0.65)	0.36 (0.24-0.53)	0.47 (0.33-0.67)
P_{trend}[a]	<0.0001	<0.0001	<0.0001
**Tumor stromal region**			
C1 (lowest)	1 (referent)	1 (referent)	1 (referent)
C2 (second)	1.09 (0.76-1.57)	1.10 (0.76-1.59)	1.17 (0.80-1.72)
C3 (third)	0.81 (0.56-1.18)	0.81 (0.56-1.17)	0.80 (0.54-1.16)
C4 (highest)	0.68 (0.47-0.98)	0.53 (0.36-0.77)	0.73 (0.51-1.05)
P_{trend}[a]	0.029	0.0012	0.054
**$CD3^+CD8^+CD45RO^-$ cell density**			
**Tumor intraepithelial region**			
C1 (lowest)	1 (referent)	1 (referent)	1 (referent)
C2 (second)	1.12 (0.73-1.71)	1.08 (0.70-1.69)	0.97 (0.64-1.47)
C3 (third)	0.78 (0.51-1.20)	0.97 (0.66-1.43)	0.68 (0.45-1.01)
C4 (highest)	0.82 (0.54-1.22)	0.61 (0.40-0.94)	0.71 (0.48-1.06)
P_{trend}[a]	0.22	0.060	0.031
**Tumor stromal region**			
C1 (lowest)	1 (referent)	1 (referent)	1 (referent)
C2 (second)	1.15 (0.78-1.69)	1.22 (0.83-1.79)	1.09 (0.73-1.60)
C3 (third)	0.88 (0.61-1.27)	0.83 (0.58-1.19)	0.94 (0.65-1.35)
C4 (highest)	1.08 (0.72-1.63)	1.00 (0.66-1.51)	0.87 (0.57-1.32)
P_{trend}[a]	0.94	0.72	0.51

[a] P_{trend} was calculated by the linear trend across the ordinal categories of the T cell densities (C1-C4, as an ordinal predictor variable) in ordinal logistic regression model for desmoplastic reaction (three categories), myxoid stroma (four categories), or keloid-like collagen bundles (four categories) at the invasive front (as an ordinal outcome variable).

Abbreviations: CI, confidence interval; OR, odds ratio; IPW, inverse probability weighting.

Supplementary Table S4. Univariable logistic regression analysis to assess the associations of macrophage with desmoplastic reaction with IPW

	Univariable OR (95% CI)		
	Immature desmoplastic reaction[a]	Myxoid stroma	Keloid-like collagen bundles
**Overall macrophage density**			
**Tumor intraepithelial region**			
C1 (lowest)	1 (referent)	1 (referent)	1 (referent)
C2 (second)	0.82 (0.52-1.29)	0.66 (0.45-0.97)	0.86 (0.58-1.29)
C3 (third)	0.93 (0.60-1.46)	0.80 (0.54-1.18)	0.89 (0.59-1.34)
C4 (highest)	0.75 (0.48-1.16)	0.58 (0.40-0.84)	0.86 (0.58-1.26)
P_{trend}[b]	0.30	0.014	0.48
**Tumor stromal region**			
C1 (lowest)	1 (referent)	1 (referent)	1 (referent)
C2 (second)	0.69 (0.44-1.07)	0.77 (0.52-1.14)	0.96 (0.64-1.44)
C3 (third)	0.68 (0.44-1.06)	0.67 (0.45-0.99)	0.99 (0.66-1.48)
C4 (highest)	0.44 (0.28-0.70)	0.50 (0.34-0.74)	1.00 (0.67-1.48)
P_{trend}[b]	0.0008	0.0004	0.98
**M1-like macrophage density**			
**Tumor intraepithelial region**			
C1 (lowest)	1 (referent)	1 (referent)	1 (referent)
C2 (second)	0.60 (0.38-0.95)	0.74 (0.51-1.08)	0.77 (0.52-1.15)
C3 (third)	0.93 (0.61-1.43)	0.78 (0.53-1.16)	0.88 (0.59-1.32)
C4 (highest)	0.63 (0.40-0.98)	0.60 (0.41-0.89)	0.69 (0.47-1.02)
P_{trend}[b]	0.16	0.019	0.12
**Tumor stromal region**			
C1 (lowest)	1 (referent)	1 (referent)	1 (referent)
C2 (second)	0.63 (0.41-0.98)	0.68 (0.46-1.01)	0.79 (0.53-1.19)
C3 (third)	0.68 (0.44-1.05)	0.66 (0.44-0.97)	0.92 (0.62-1.36)
C4 (highest)	0.45 (0.29-0.71)	0.48 (0.33-0.70)	0.70 (0.47-1.04)
P_{trend}[b]	0.0014	0.0002	0.15
**M2-like macrophage density**			
**Tumor intraepithelial region**			
C1 (lowest)	1 (referent)	1 (referent)	1 (referent)
C2 (second)	1.00 (0.64-1.57)	1.21 (0.82-1.78)	1.15 (0.76-1.74)
C3 (third)	0.97 (0.62-1.53)	0.96 (0.65-1.42)	1.08 (0.73-1.62)
C4 (highest)	0.90 (0.57-1.40)	0.89 (0.60-1.32)	1.13 (0.75-1.69)
P_{trend}[b]	0.62	0.34	0.65
**Tumor stromal region**			
C1 (lowest)	1 (referent)	1 (referent)	1 (referent)
C2 (second)	1.12 (0.71-1.75)	1.09 (0.73-1.61)	1.24 (0.82-1.87)
C3 (third)	1.03 (0.65-1.62)	1.12 (0.75-1.65)	1.62 (1.10-2.39)
C4 (highest)	0.94 (0.60-1.48)	0.94 (0.64-1.38)	1.47 (1.00-2.18)
P_{trend}[b]	0.70	0.80	0.024

[a] To avoid violation of the proportional odds assumption, the binary categories was used for desmoplastic rection (immature vs. intermediate/mature).
[b] P_{trend} was calculated by the linear trend across the ordinal categories of the macrophage densities (C1-C4, as an ordinal predictor variable) in ordinal logistic regression model for desmoplastic reaction (binary categories), myxoid stroma (four categories), or keloid-like collagen bundles (four categories) at the invasive front (as an ordinal outcome variable).
Abbreviations: CI, confidence interval; OR, odds ratio; IPW, inverse probability weighting.

Supplementary Table S5. Univariable and multivariable logistic regression analysis to assess the associations of T-cell with desmoplastic reaction including only pT3 and pT4 cases with IPW

	Immature desmoplastic reaction	
	Univariable OR (95% CI)	Multivariable OR[a] (95% CI)
**$CD3^+$ cell density**		
**Tumor intraepithelial region**		
C1 (lowest)	1 (referent)	1 (referent)
C2 (second)	0.68 (0.43-1.09)	0.71 (0.44-1.13)
C3 (third)	0.58 (0.37-0.92)	0.61 (0.38-0.98)
C4 (highest)	0.64 (0.41-1.02)	0.71 (0.44-1.13)
P_{trend}[b]	0.041	0.10
**Tumor stromal region**		
C1 (lowest)	1 (referent)	1 (referent)
C2 (second)	0.96 (0.61-1.53)	0.79 (0.54-1.16)
C3 (third)	0.68 (0.43-1.08)	0.52 (0.35-0.77)
C4 (highest)	0.89 (0.56-1.40)	0.50 (0.34-0.73)
P_{trend}[b]	0.35	0.37
**$CD3^+CD4^+$ cell density**		
**Tumor intraepithelial region**		
C1 (lowest)	1 (referent)	1 (referent)
C2 (second)	0.72 (0.45-1.14)	0.72 (0.45-1.13)
C3 (third)	0.56 (0.37-0.87)	0.59 (0.38-0.92)
C4 (highest)	0.82 (0.53-1.29)	0.85 (0.85-1.34)
P_{trend}[b]	0.19	0.27
**Tumor stromal region**		
C1 (lowest)	1 (referent)	1 (referent)
C2 (second)	1.07 (0.67-1.70)	1.03 (0.70-1.50)
C3 (third)	0.83 (0.52-1.32)	0.73 (0.50-1.08)
C4 (highest)	0.84 (0.54-1.31)	0.56 (0.38-0.82)
P_{trend}[b]	0.29	0.35
**$CD3^+CD8^+$ cell density**		
**Tumor intraepithelial region**		
C1 (lowest)	1 (referent)	1 (referent)
C2 (second)	0.62 (0.41-0.93)	0.79 (0.50-1.24)
C3 (third)	0.70 (0.47-1.05)	0.64 (0.40-1.03)
C4 (highest)	0.65 (0.43-0.97)	0.72 (0.72-1.11)
P_{trend}[b]	0.024	0.054
**Tumor stromal region**		
C1 (lowest)	1 (referent)	1 (referent)
C2 (second)	1.01 (0.65-1.57)	1.06 (0.68-1.65)
C3 (third)	0.76 (0.49-1.18)	0.77 (0.49-1.19)
C4 (highest)	0.83 (0.53-1.28)	0.82 (0.53-1.27)
P_{trend}[b]	0.24	0.22
**$CD3^+CD4^+FOXP3^+$ cell density**		
**Tumor intraepithelial region**		
C1 (lowest)	1 (referent)	1 (referent)
C2 (second)	1.65 (0.97-2.79)	1.71 (1.04-2.83)
C3 (third)	0.93 (0.55-1.57)	0.98 (0.57-1.69)
C4 (highest)	1.06 (0.62-1.80)	1.11 (0.63-1.95)
P_{trend}[b]	0.78	0.62
**Tumor stromal region**		
C1 (lowest)	1 (referent)	1 (referent)
C2 (second)	0.76 (0.49-1.19)	0.77 (0.48-1.21)
C3 (third)	0.68 (0.43-1.08)	0.77 (0.49-1.22)
C4 (highest)	1.04 (0.66-1.65)	1.12 (0.70-1.79)
P_{trend}[b]	0.58	0.92

**CD3⁺CD4⁺CD45RO⁺ cell density**		
**Tumor intraepithelial region**		
C1 (lowest)	1 (referent)	1 (referent)
C2 (second)	0.66 (0.42-1.04)	0.69 (0.44-1.09)
C3 (third)	0.58 (0.37-0.89)	0.64 (0.41-0.99)
C4 (highest)	0.62 (0.40-0.96)	0.65 (0.42-1.02)
P_{trend} [b]	0.015	0.034
**Tumor stromal region**		
C1 (lowest)	1 (referent)	1 (referent)
C2 (second)	1.15 (0.72-1.83)	1.23 (0.77-1.96)
C3 (third)	0.90 (0.57-1.40)	0.96 (0.61-1.51)
C4 (highest)	0.75 (0.48-1.17)	0.76 (0.48-1.18)
P_{trend} [b]	0.14	0.15
**CD3⁺CD4⁺CD45RO⁻ cell density**		
**Tumor intraepithelial region**		
C1 (lowest)	1 (referent)	1 (referent)
C2 (second)	1.41 (0.88-2.23)	1.45 (0.91-2.30)
C3 (third)	0.86 (0.56-1.33)	0.88 (0.56-1.37)
C4 (highest)	1.40 (0.86-2.28)	1.42 (0.87-2.33)
P_{trend} [b]	0.36	0.33
**Tumor stromal region**		
C1 (lowest)	1 (referent)	1 (referent)
C2 (second)	0.81 (0.52-1.26)	0.83 (0.53-1.31)
C3 (third)	0.93 (0.59-1.46)	1.04 (0.66-1.63)
C4 (highest)	1.06 (0.69-1.62)	1.08 (0.70-1.67)
P_{trend} [b]	0.86	0.68
**CD3⁺CD8⁺CD45RO⁺ cell density**		
**Tumor intraepithelial region**		
C1 (lowest)	1 (referent)	1 (referent)
C2 (second)	0.52 (0.33-0.82)	0.54 (0.34-0.85)
C3 (third)	0.46 (0.30-0.73)	0.49 (0.31-0.78)
C4 (highest)	0.56 (0.37-0.85)	0.61 (0.39-0.95)
P_{trend} [b]	0.0005	0.0025
**Tumor stromal region**		
C1 (lowest)	1 (referent)	1 (referent)
C2 (second)	1.17 (0.76-1.80)	1.21 (0.78-1.88)
C3 (third)	0.84 (0.54-1.31)	0.91 (0.57-1.43)
C4 (highest)	0.78 (0.50-1.21)	0.81 (0.52-1.26)
P_{trend} [b]	0.21	0.34
**CD3⁺CD8⁺CD45RO⁻ cell density**		
**Tumor intraepithelial region**		
C1 (lowest)	1 (referent)	1 (referent)
C2 (second)	1.30 (0.79-2.14)	1.39 (0.82-2.37)
C3 (third)	0.75 (0.45-1.24)	0.77 (0.46-1.29)
C4 (highest)	0.96 (0.59-1.57)	1.05 (0.64-1.73)
P_{trend} [b]	0.63	0.89
**Tumor stromal region**		
C1 (lowest)	1 (referent)	1 (referent)
C2 (second)	1.15 (0.72-1.82)	1.24 (0.77-2.00)
C3 (third)	0.80 (0.52-1.23)	0.76 (0.49-1.19)
C4 (highest)	1.33 (0.81-2.18)	1.39 (0.84-2.29)
P_{trend} [b]	0.57	0.54

[a] The multivariable ordinal logistic regression model initially included age, sex, year of diagnosis, family history of colorectal cancer, tumor location, tumor grade, microsatellite instability, CpG island methylator phenotype, long-interspersed nucleotide element-1 methylation level, *KRAS*, *BRAF*, and *PIK3CA* mutations. A backward elimination with a threshold P of 0.05 was used to select variables for the final model.

[b] P_{trend} was calculated by the linear trend across the ordinal categories of the T cell densities (C1-C4, as an ordinal predictor variable) in ordinal logistic regression model for desmoplastic reaction (three categories), myxoid stroma (four categories), or keloid-like collagen bundles (four categories) at the invasive front (as an ordinal outcome variable).

Abbreviations: CI, confidence interval; OR, odds ratio; IPW, inverse probability weighting.

Supplementary Table S6. Univariable and multivariable logistic regression analysis to assess the associations of macrophage with desmoplastic reaction including only pT3 and pT4 cases with IPW

	Immature desmoplastic reaction[a]	
	Univariable OR (95% CI)	Multivariable OR[b] (95% CI)
**Overall macrophage density**		
**Tumor intraepithelial region**		
C1 (lowest)	1 (referent)	1 (referent)
C2 (second)	0.75 (0.45-1.27)	0.73 (0.43-1.23)
C3 (third)	0.81 (0.48-1.36)	0.83 (0.48-1.43)
C4 (highest)	0.77 (0.46-1.28)	0.76 (0.44-1.32)
P_{trend}[c]	0.37	0.41
**Tumor stromal region**		
C1 (lowest)	1 (referent)	1 (referent)
C2 (second)	0.61 (0.37-1.01)	0.66 (0.39-1.12)
C3 (third)	0.64 (0.38-1.06)	0.68 (0.40-1.16)
C4 (highest)	0.42 (0.25-0.70)	0.42 (0.24-0.72)
P_{trend}[c]	0.0020	0.0036
**M1-like macrophage density**		
**Tumor intraepithelial region**		
C1 (lowest)	1 (referent)	1 (referent)
C2 (second)	0.58 (0.35-0.96)	0.57 (0.34-0.97)
C3 (third)	0.91 (0.56-1.48)	0.98 (0.60-1.61)
C4 (highest)	0.72 (0.44-1.17)	0.77 (0.46-1.31)
P_{trend}[c]	0.31	0.53
**Tumor stromal region**		
C1 (lowest)	1 (referent)	1 (referent)
C2 (second)	0.64 (0.39-1.07)	0.71 (0.42-1.20)
C3 (third)	0.58 (0.35-0.97)	0.67 (0.40-1.13)
C4 (highest)	0.43 (0.26-0.72)	0.46 (0.27-0.79)
P_{trend}[c]	0.0015	0.0056
**M2-like macrophage density**		
**Tumor intraepithelial region**		
C1 (lowest)	1 (referent)	1 (referent)
C2 (second)	0.82 (0.49-1.38)	0.76 (0.45-1.30)
C3 (third)	0.75 (0.45-1.26)	0.69 (0.41-1.18)
C4 (highest)	0.86 (0.52-1.42)	0.81 (0.47-1.40)
P_{trend}[c]	0.49	0.40
**Tumor stromal region**		
C1 (lowest)	1 (referent)	1 (referent)
C2 (second)	0.94 (0.57-1.58)	0.98 (0.57-1.67)
C3 (third)	0.80 (0.47-1.34)	0.78 (0.46-1.32)
C4 (highest)	0.84 (0.50-1.40)	0.83 (0.49-1.42)
P_{trend}[c]	0.40	0.37

[a] To avoid violation of the proportional odds assumption, the binary categories was used for desmoplastic rection (immature vs. intermediate/mature).

[b] The multivariable ordinal logistic regression model initially included age, sex, year of diagnosis, family history of colorectal cancer, tumor location, tumor grade, microsatellite instability, CpG island methylator phenotype, long-interspersed nucleotide element-1 methylation level, *KRAS*, *BRAF*, and *PIK3CA* mutations. A backward elimination with a threshold P of 0.05 was used to select variables for the final model.

[c] P_{trend} was calculated by the linear trend across the ordinal categories of the macrophage densities (C1-C4, as an ordinal predictor variable) in ordinal logistic regression model for desmoplastic reaction (binary categories), myxoid stroma (four categories), or keloid-like collagen bundles (four categories) at the invasive front (as an ordinal outcome variable).

Abbreviations: CI, confidence interval; OR, odds ratio; IPW, inverse probability weighting.

Supplementary table S7. IPW-adjusted logistic regression analysis between T-cell and immature desmoplastic reaction stratified by MSI

	Immature desmoplastic reaction		Myxoid stroma		Keloid-like collagen bundles	
	Univariable OR (95% CI)[b,c]	Multivariable OR (95% CI)[b,c,d]	Univariable OR (95% CI)[b,c]	Multivariable OR (95% CI)[b,c,d]	Univariable OR (95% CI)[b,c]	Multivariable OR (95% CI)[b,c,d]
Intraepithelial $CD3^+CD8^+CD45RO^+$ cell density (cells/mm^2)[a]						
**Non-MSI-high**						
C1 (zero)	1 (referent)	1 (referent)	1 (referent)	1 (referent)	1 (referent)	1 (referent)
C2 (low)	0.58 (0.38-0.89)	0.56 (0.37-0.85)	0.63 (0.41-0.94)	0.59 (0.39-0.89)	0.64 (0.42-0.97)	0.62 (0.42-0.94)
C3 (intermediate)	0.49 (0.32-0.75)	0.49 (0.32-0.75)	0.57 (0.39-0.83)	0.54 (0.37-0.80)	0.52 (0.34-0.79)	0.51 (0.33-0.78)
C4 (high)	0.49 (0.32-0.77)	0.50 (0.32-0.78)	0.37 (0.23-0.59)	0.36 (0.23-0.58)	0.45 (0.29-0.70)	0.45 (0.29-0.70)
**MSI-high**						
C1 (zero)	1 (referent)	1 (referent)	1 (referent)	1 (referent)	1 (referent)	1 (referent)
C2 (low)	0.43 (0.17-1.10)	0.55 (0.22-1.39)	0.58 (0.24-1.41)	0.71 (0.30-1.70)	0.44 (0.19-1.02)	0.51 (0.22-1.15)
C3 (intermediate)	0.75 (0.31-1.85)	0.90 (0.34-2.36)	0.58 (0.21-1.57)	0.62 (0.23-1.69)	0.60 (0.24-1.52)	0.65 (0.25-1.69)
C4 (high)	0.38 (0.18-0.82)	0.39 (0.18-0.88)	0.37 (0.16-0.84)	0.36 (0.16-0.85)	0.47 (0.23-0.95)	0.47 (0.23-0.94)
$P_{interaction}$[e]	0.99	0.88	0.99	0.96	0.62	0.63
Stroma M1-like macrophage density (cells/mm^2)						
**Non-MSI-high**						
C1 (lowest)	1 (referent)	1 (referent)	1 (referent)	1 (referent)	1 (referent)	1 (referent)
C2 (second)	0.75 (0.49-1.14)	0.79 (0.52-1.21)	0.69 (0.45-1.05)	0.73 (0.48-1.11)	0.85 (0.55-1.33)	0.87 (0.56-1.35)
C3 (third)	0.82 (0.54-1.26)	0.91 (0.60-1.40)	0.68 (0.44-1.04)	0.73 (0.48-1.13)	1.04 (0.68-1.59)	1.07 (0.70-1.65)
C4 (highest)	0.57 (0.37-0.89)	0.57 (0.37-0.90)	0.52 (0.34-0.80)	0.52 (0.34-0.79)	0.75 (0.48-1.18)	0.75 (0.48-1.18)
**MSI-high**						
C1 (lowest)	1 (referent)	1 (referent)	1 (referent)	1 (referent)	1 (referent)	1 (referent)
C2 (second)	0.80 (0.28-2.26)	1.28 (0.40-4.06)	0.87 (0.26-2.90)	1.25 (0.35-4.54)	0.57 (0.22-1.44)	0.81 (0.30-2.22)
C3 (third)	0.60 (0.22-1.66)	0.97 (0.32-2.98)	0.61 (0.19-1.96)	0.91 (0.26-3.12)	0.39 (0.14-1.09)	0.54 (0.19-1.58)
C4 (highest)	0.50 (0.20-1.24)	0.70 (0.25-1.92)	0.46 (0.15-1.34)	0.56 (0.18-1.78)	0.47 (0.19-1.16)	0.62 (0.24-1.59)
$P_{interaction}$[e]	0.63	0.88	0.66	0.76	0.33	0.51

[a] As intraepithelial $CD3^+CD8^+CD45RO^+$ cell densities were 0/mm^2 (consisting the largest category C1), the remaining cases were divided into tertiles according to density (C2-C4).

[b] IPW was applied to reduce a bias due to the availability of tumor tissue after cancer diagnosis (see "Statistical analysis" section for details).

[c] ORs were estimated for each stratum on the basis of the cases with intraepithelial T cell/stromal M1-like macrophage densities, using a re-parameterization of the interaction term in a single regression model for the stratified analyses.

[d] The multivariable ordinal logistic regression model initially included age, sex, year of diagnosis, family history of colorectal cancer, tumor location, tumor grade, microsatellite instability, CpG island methylator phenotype, long-interspersed nucleotide element-1 methylation level, *KRAS*, *BRAF*, and *PIK3CA* mutations. A backward elimination with a threshold P of 0.05 was used to select variables for the final model.

[e] $P_{interaction}$ was calculated using the Wald test for the cross-product of the density of immune cells (four ordinal categories: C1-C4) and MSI status (high vs. non-high) in the IPW-adjusted ordinal logistic regression model.

Abbreviations: CI, confidence interval; OR, odds ratio; MSI, microsatellite instability; IPW, inverse probability weighting.

Supplementary Table S8. Desmoplastic reaction and its components including only pT3 and pT4 cases and patient survival with IPW

		Colorectal cancer-specific survival[a]			Overall survival[a]		
	No. of cases	No. of events	Univariable HR (95% CI)*	Multivariable HR (95% CI)[b]	No. of events	Univariable HR (95% CI)*	Multivariable HR (95% CI)[b]
Desmoplastic reaction							
Immature	8	3	1 (referent)	1 (referent)	4	1 (referent)	1 (referent)
Intermediate	34	3	0.19 (0.04-0.87)	0.33 (0.10-1.08)	7	0.33 (0.10-1.08)	0.28 (0.10-0.82)
Mature	150	12	0.21 (0.07-0.68)	0.57 (0.22-1.49)	45	0.57 (0.22-1.49)	0.36 (0.14-0.91)
P_{trend}[c]			0.11	0.87		0.87	0.37
Myxoid stroma							
C1 (marked)	2	1	1 (referent)	1 (referent)	1	1 (referent)	1 (referent)
C2 (moderate)	6	2	0.48 (0.04-5.24)	0.11 (0.01-2.31)	3	0.60 (0.04-8.25)	1.58 (0.07-35.2)
C3 (mild)	77	11	0.21 (0.02-1.86)	0.10 (0.01-1.10)	22	0.37 (0.03-4.42)	0.71 (0.04-12.5)
C4 (absent)	107	4	0.06 (0.01-0.57)	0.02 (0.002-0.25)	30	0.33 (0.03-4.00)	0.66 (0.04-11.6)
P_{trend}[c]			0.0003	0.0006		0.32	0.40
Keloid-like collagen bundles							
C1 (marked)	8	2	1 (referent)	1 (referent)	3	1 (referent)	1 (referent)
C2 (moderate)	32	3	0.31 (0.05-1.93)	0.07 (0.01-0.48)	7	0.54 (0.13-2.22)	1.16 (0.31-4.31)
C3 (mild)	119	12	0.44 (0.17-1.99)	0.15 (0.03-0.93)	36	0.93 (0.27-3.21)	1.17 (0.37-3.68)
C4 (absent)	33	1	0.04 (0.04-0.94)	0.03 (0.002-0.29)	10	0.69 (0.17-2.73)	1.34 (0.35-5.10)
P_{trend}[c]			0.11	0.21		0.86	0.66

[a] IPW was applied to reduce a bias due to the availability of tumor tissue after cancer diagnosis (see "Statistical Analysis" subsection for details).
[b] The multivariable Cox regression model initially included sex, age, year of diagnosis, family history of colorectal cancer, tumor location, tumor grade, microsatellite instability, CpG island methylator phenotype, long-interspersed nucleotide element-1 methylation level, *KRAS*, *BRAF*, and *PIK3CA* mutations, tumor-infiltrating lymphocytes, intratumoral periglandular reaction, peritumoral lymphocytic reaction, Crohn's-like lymphoid reaction, intraepithelial $CD3^+CD8^+CD45RO^+$ T cell density, and stroma M1-like macrophage density. A backward elimination with a threshold P of 0.05 was used to select variables for the final models.
[c] P_{trend} value was calculated by the linear trend across the ordinal categories of the desmoplastic reaction, myxoid stroma, and keloid-like collagen bundles in the IPW-adjusted Cox regression model.
Abbreviations: CI, confidence interval; HR, hazard ratio; IPW, inverse probability weighting.

Supplementary Table S9. Desmoplastic reaction and patient survival according to $CD3^+CD8^+CD45RO^+$ T-cell in intraepithelial region with IPW

		Colorectal cancer-specific survival[a,b]			Overall survival[a,b]		
	No. of cases	No. of events	Univariable HR (95% CI)	Multivariable HR (95% CI)[d]	No. of events	Univariable HR (95% CI)	Multivariable HR (95% CI)[d]
**Low $CD3^+CD8^+CD45RO^+$ cell density in intraepithelial region[c]**							
Desmoplastic reaction							
Immature	163	91	1 (referent)	1 (referent)	105	1 (referent)	1 (referent)
Intermediate	119	37	0.41 (0.28-0.61)	0.56 (0.39-0.81)	58	0.51 (0.36-0.73)	0.61 (0.43-0.88)
Mature	170	29	0.18 (0.12-0.28)	0.28 (0.18-0.45)	60	0.30 (0.21-0.43)	0.40 (0.27-0.58)
**High $CD3^+CD8^+CD45RO^+$ cell density in intraepithelial region[c]**							
Desmoplastic reaction							
Immature	104	49	1 (referent)	1 (referent)	63	1 (referent)	1 (referent)
Intermediate	111	28	0.45 (0.27-0.73)	0.62 (0.37-1.04)	42	0.49 (0.32-0.75)	0.58 (0.38-0.89)
Mature	237	31	0.21 (0.13-0.33)	0.34 (0.20-0.56)	96	0.46 (0.33-0.64)	0.57 (0.40-0.81)
$P_{interaction}$[e]			0.66	0.62		0.071	0.12
**Low $CD3^+CD8^+CD45RO^+$ cell density in intraepithelial region[c]**							
Myxoid stroma							
Marked	90	58	1 (referent)	1 (referent)	63	1 (referent)	1 (referent)
Moderate	73	33	0.57 (0.37-0.87)	0.71 (0.46-1.09)	42	0.58 (0.38-0.89)	0.65 (0.40-1.05)
Mild	199	52	0.25 (0.17-0.37)	0.42 (0.28-0.61)	86	0.32 (0.23-0.46)	0.44 (0.30-0.63)
Absent	90	14	0.14 (0.08-0.27)	0.22 (0.12-0.43)	32	0.26 (0.16-0.42)	0.34 (0.20-0.57)
**High $CD3^+CD8^+CD45RO^+$ cell density in intraepithelial region[c]**							
Myxoid stroma							
Marked	40	19	1 (referent)	1 (referent)	25	1 (referent)	1 (referent)
Moderate	64	30	0.87 (0.48-1.58)	0.69 (0.36-1.31)	38	0.88 (0.51-1.49)	0.86 (0.49-1.49)
Mild	210	47	0.34 (0.20-0.58)	0.42 (0.23-0.77)	86	0.45 (0.28-0.71)	0.52 (0.32-0.84)
Absent	138	12	0.14 (0.07-0.30)	0.22 (0.10-0.47)	52	0.41 (0.25-0.67)	0.51 (0.31-0.86)
$P_{interaction}$[e]			0.97	0.85		0.17	0.23
**Low $CD3^+CD8^+CD45RO^+$ cell density in intraepithelial region[c]**							
Keloid-like collagen bundles							
Marked	87	53	1 (referent)	1 (referent)	64	1 (referent)	1 (referent)
Moderate	174	64	0.43 (0.30-0.62)	0.52 (0.36-0.75)	87	0.44 (0.31-0.62)	0.51 (0.35-0.74)
Mild	160	37	0.23 (0.15-0.35)	0.36 (0.25-0.54)	63	0.29 (0.20-0.42)	0.42 (0.29-0.61)
Absent	31	3	0.07 (0.02-0.25)	0.08 (0.02-0.33)	9	0.15 (0.07-0.32)	0.17 (0.08-0.37)

**High CD3$^+$CD8$^+$CD45RO$^+$ cell density in intraepithelial region**[c]							
Keloid-like collagen bundles							
Marked	41	17	1 (referent)	1 (referent)	23	1 (referent)	1 (referent)
Moderate	161	55	0.79 (0.45-1.39)	0.66 (0.38-1.13)	75	0.77 (0.47-1.24)	0.68 (0.43-1.08)
Mild	209	33	0.31 (0.17-0.56)	0.36 (0.20-0.65)	87	0.55 (0.34-0.87)	0.59 (0.37-0.93)
Absent	41	3	0.12 (0.04-0.39)	0.13 (0.04-0.45)	16	0.44 (0.23-0.83)	0.44 (0.24-0.79)
$P_{interaction}$[e]			0.51	0.92		0.011	0.058

[a] IPW was applied to reduce a bias due to the availability of tumor tissue after cancer diagnosis (see "Statistical analysis" subsection for details).
[b] HRs were estimated for each stratum on the basis of the cases with myxoid stroma using a re-parameterization of the interaction term in a single Cox regression model for the stratified analyses.
[c] T cell density was graded as a binary category (fiftieth-percentile, low and high).
[d] The multivariable ordinal logistic regression model initially included age, sex, year of diagnosis, family history of colorectal cancer, tumor location, tumor grade, AJCC disease stage, microsatellite instability, CpG island methylator phenotype, long-interspersed nucleotide element-1 methylation level, *KRAS*, *BRAF*, *PIK3CA* mutations, tumor-infiltrating lymphocytes, intratumoral periglandular reaction, peritumoral lymphocytic reaction, Crohn's-like lymphoid reaction, and stroma M1-like macrophage densities. A backward elimination with a threshold P of 0.05 was used to select variables for the final models.
[e] $P_{interaction}$ (two-sided) was calculated using the Wald test for the cross-product of desmoplastic reaction (immature, intermediate, and mature), myxoid stroma grade and keloid-like collagen bundles grade (marked, moderate, mild, and absent) and density of CD3$^+$CD8$^+$CD45RO$^+$ T cell (low vs. high) in the IPW-adjusted Cox regression model.
Abbreviations: IPW, inverse probability weighting; CI, confidence interval; HR, hazard ratio.

Supplementary Table S10. Desmoplastic reaction and patient survival according to M1-like macrophage in stroma region with IPW

		Colorectal cancer-specific survival[a,b]			Overall survival[a,b]		
	No. of cases	No. of events	Univariable HR (95% CI)	Multivariable HR (95% CI)[d]	No. of events	Univariable HR (95% CI)	Multivariable HR (95% CI)[d]
**Low M1-like macrophage density in tumor stromal region**[c]							
Desmoplastic reaction							
Immature	158	99	1 (referent)	1 (referent)	116	1 (referent)	1 (referent)
Intermediate	102	39	0.41 (0.28-0.60)	0.55 (0.39-0.79)	55	0.46 (0.32-0.65)	0.56 (0.38-0.79)
Mature	190	32	0.16 (0.10-0.24)	0.22 (0.14-0.34)	70	0.25 (0.18-0.35)	0.31 (0.25-0.49)
**High M1-like macrophage density in tumor stromal region**[c]							
Desmoplastic reaction							
Immature	117	48	1 (referent)	1 (referent)	60	1 (referent)	1 (referent)
Intermediate	123	27	0.48 (0.29-0.79)	0.71 (0.43-1.16)	45	0.61 (0.41-0.93)	0.75 (0.49-1.14)
Mature	210	28	0.26 (0.16-0.43)	0.50 (0.29-0.85)	83	0.59 (0.42-0.85)	0.86 (0.57-1.28)
$P_{interaction}$[e]			0.14	0.024		0.0005	0.0002
**Low M1-like macrophage density in tumor stromal region**[c]							
Myxoid stroma							
Marked	80	50	1 (referent)	1 (referent)	59	1 (referent)	1 (referent)
Moderate	78	49	0.93 (0.64-1.37)	1.03 (0.70-1.53)	57	0.90 (0.62-1.33)	1.01 (0.68-1.50)
Mild	195	55	0.29 (0.20-0.43)	0.42 (0.29-0.63)	92	0.35 (0.25-0.50)	0.46 (0.32-0.65)
Absent	97	16	0.14 (0.08-0.24)	0.21 (0.11-0.37)	33	0.23 (0.14-0.37)	0.30 (0.19-0.50)
**High M1-like macrophage density in tumor stromal region**[c]							
Myxoid stroma							
Marked	49	26	1 (referent)	1 (referent)	29	1 (referent)	1 (referent)
Moderate	68	22	0.47 (0.26-0.85)	0.45 (0.25-0.81)	31	0.56 (0.33-0.97)	0.48 (0.27-0.85)
Mild	205	45	0.28 (0.17-0.47)	0.43 (0.26-0.71)	79	0.42 (0.27-0.67)	0.48 (0.31-0.77)
Absent	128	10	0.13 (0.06-0.28)	0.26 (0.12-0.55)	49	0.43 (0.27-0.71)	0.59 (0.36-0.96)
$P_{interaction}$[e]			0.96	0.52		0.016	0.015
**Low M1-like macrophage density in tumor stromal region**[c]							
Keloid-like collagen bundles							
Marked	62	43	1 (referent)	1 (referent)	50	1 (referent)	1 (referent)
Moderate	182	86	0.55 (0.38-0.79)	0.60 (0.42-0.84)	110	0.56 (0.39-0.81)	0.61 (0.43-0.87)
Mild	169	37	0.20 (0.13-0.32)	0.28 (0.18-0.43)	71	0.29 (0.19-0.43)	0.35 (0.24-0.52)
Absent	37	4	0.07 (0.02-0.20)	0.08 (0.03-0.25)	10	0.13 (0.07-0.27)	0.16 (0.08-0.31)

**High M1-like macrophage density in tumor stromal region**[c]							
Keloid-like collagen bundles							
Marked	66	28	1 (referent)	1 (referent)	38	1 (referent)	1 (referent)
Moderate	156	40	0.50 (0.30-0.83)	0.63 (0.38-1.04)	59	0.51 (0.33-0.78)	0.57 (0.36-0.90)
Mild	196	33	0.29 (0.17-0.49)	0.53 (0.31-0.93)	78	0.49 (0.32-0.73)	0.72 (0.46-1.11)
Absent	32	2	0.09 (0.02-0.39)	0.21 (0.05-0.93)	13	0.46 (0.25-0.86)	0.61 (0.33-1.12)
$P_{interaction}$[e]			0.30	0.029		0.0040	0.0001

[a] IPW was applied to reduce a bias due to the availability of tumor tissue after cancer diagnosis (see “Statistical analysis” subsection for details).

[b] HRs were estimated for each stratum on the basis of the cases with myxoid stroma using a re-parameterization of the interaction term in a single Cox regression model for the stratified analyses.

[c] T cell density was graded as a binary category (fiftieth-percentile, low and high).

[d] The multivariable ordinal logistic regression model initially included age, sex, year of diagnosis, family history of colorectal cancer, tumor location, tumor grade, AJCC disease stage, microsatellite instability, CpG island methylator phenotype, long-interspersed nucleotide element-1 methylation level, *KRAS*, *BRAF*, *PIK3CA* mutations, tumor-infiltrating lymphocytes, intratumoral periglandular reaction, peritumoral lymphocytic reaction, Crohn's-like lymphoid reaction, and intraepithelial $CD3^+CD8^+CD45RO^+$ T cell densities. A backward elimination with a threshold P of 0.05 was used to select variables for the final models.

[e] $P_{interaction}$ (two-sided) was calculated using the Wald test for the cross-product of desmoplastic reaction (immature, intermediate, and mature), myxoid stroma grade, and keloid-like collagen bundles grade (marked, moderate, mild, and absent) and density of M1-like macrophage (low vs. high) in the IPW-adjusted Cox regression model.

Abbreviations: IPW, inverse probability weighting; CI, confidence interval; HR, hazard ratio.
